# Chronic stress drives ovarian cancer progression via myeloid-derived suppressor cells infiltration and Notch signaling pathway activation

**DOI:** 10.3389/fimmu.2025.1593299

**Published:** 2025-12-19

**Authors:** Yadiel A. Rivera-López, Alanis P. Torres-Rosado, Jaydiel A. Casiano-Martínez, Luinet L. Meléndez-Rodríguez, Raian Imad-Hamad, Sofía M. Hernández-Carrasquillo, Luis M. Rivera-Pérez, Melanie Ortiz-León, Orlando I. Torres-Rodríguez, Alexandra N. Aquino-Acevedo, Yesenia Castillo-Ocampo, Grace Duffey, Jaileene Pérez-Morales, Mary K. Townsend, Lauren C. Peres, Paulo C. Rodriguez, Shelley S. Tworoger, Guillermo N. Armaiz-Pena

**Affiliations:** 1 Department of Basic Sciences, Division of Pharmacology, School of Medicine, Ponce Health Sciences University, Ponce, Puerto Rico; 2 Department of Biomedical Sciences, School of Dental Medicine, Ponce Health Sciences University, Ponce, Puerto Rico; 3 Department of Biology, University of Puerto Rico, Mayagüez, Puerto Rico; 4 Department of Allied Health, University of Puerto Rico, Ponce, Puerto Rico; 5 Division of Oncological Sciences, Knight Cancer Institute, Oregon Health and Science University, Portland, OR, United States; 6 Department of Cancer Epidemiology, Moffitt Cancer Center and Research Institute, Tampa, FL, United States; 7 Department of Immunology, Moffitt Cancer Center and Research Institute, Tampa, FL, United States; 8 Divisions of Cancer Biology, Mental Health, and Women’s Health, Ponce Research Institute, Ponce, Puerto Rico

**Keywords:** chronic stress, corticosterone, epinephrine, GSK3β, MDSCs, norepinephrine, Notch, ovarian cancer

## Abstract

**Background:**

Ovarian cancer (OC) is a leading cause of cancer-related death among women, with elevated levels of stress hormones linked to OC progression and immune evasion in the tumor microenvironment (TME). Chronic psychosocial stress has been associated with the expansion of immunosuppressive myeloid-derived suppressor cells (MDSCs), which promote tumor growth and negatively impact patient outcomes. This study tested how chronic daily restraint stress affected ovarian cancer progression, Notch signaling, and MDSCs in the OC TME. We hypothesized that chronic stress increases MDSCs infiltration in the TME, enhances Notch signaling in OC cells, and promotes cancer progression.

**Methods:**

Female C57BL/6 mice were injected with ID8^Luc^ or IG10^Luc^ OC cells and subjected to daily restraint stress. We also isolated bone marrow from naïve C57BL/6 mice to differentiate myeloid cell precursors into MDSCs. These cells were then exposed to norepinephrine (NE), epinephrine (EPI), or corticosterone (CC). To further evaluate the effects of stress hormones on the dysregulation of the Notch signaling pathway, we treated OC cells with NE, EPI, or CC.

**Results:**

Chronic daily restraint stress increased MDSCs infiltration and enriched polymorphonuclear (PMN)-MDSCs in the TME and bone marrow in both models. *Ex vivo* studies demonstrated an increased enrichment of PMN-MDSCs along with a reduction of mononuclear (M)-MDSCs in the groups treated with stress hormones, particularly CC. Our results showed that stress hormones significantly increased the expression of notch intracellular domain (NICD) in OC cells. Additionally, we observed increased mRNA levels of Notch1, Jagged2, and Hes1, along with elevated NICD and HES1 protein levels, mediated by CC-induced GSK3β phosphorylation. Pharmacological inhibition of NICD and g^Luc^ocorticoid receptors blocked the CC-induced Notch pathway activation *via* GSK3β phosphorylation. Moreover, tumors from mice subjected to restraint stress had elevated expression of *Notch1*, *Jagged 2*, NICD, *HES1*, GR, ADRB2, and pS9-GSK3β.

**Conclusion:**

These data indicate that chronic stress leads to MDSCs infiltration and suppressive activity, which contributes to an immunosuppressive TME and OC progression.

## Highlights

- Chronic restraint stress led to the enrichment, accumulation, and polarization of myeloid-derived suppressor cells (MDSCs) in the bone marrow and tumors from ovarian cancer mouse models.
- Stress hormones enhance the suppressive activity of MDSCs on CD8+ T-cells.
- Chronic restraint stress activated the Notch signaling pathway in tumors from ovarian cancer mouse models.
- Corticosterone triggered the Notch signaling pathway *via* GSK3β in ovarian cancer cells.
- Pharmacological inhibition with DAPT and RU-486 prevented corticosterone-induced activation of the Notch pathway through GSK3β.

## 1 Introduction

Approximately 324,603 women will receive a new diagnosis of ovarian cancer (OC), while 206,956 will die from this disease worldwide, with five-year survival rates remaining below 50% (1, 2). The absence of early detection methods and subsequent late diagnoses leads to poor treatment outcomes for women with OC (3, 4). Cancer outcomes can be influenced by exposure to traumatic events, such as a cancer diagnosis and treatment, which can lead to behavioral disorders like chronic stress (5–7). Patients with OC experience higher levels of anxiety and depression (8). Moreover, chronic stress and other behavioral disorders are associated with OC progression (5, 9). Although 75% to 90% of human diseases are linked to dysregulation of the stress response, the connections and mechanisms between stress exposure and the pathophysiological processes underlying disease are still poorly understood (10). For instance, prolonged exposure to chronic stress can impact immune system function, significantly increasing tumor-associated inflammation and facilitating immune evasion in various diseases, including OC (8, 9).

Tumor-infiltrating myeloid cells, including myeloid-derived suppressor cells (MDSCs), are linked to poor outcomes in patients with cancer (11, 12). MDSCs, which include polymorphonuclear (PMN-MDSCs) and mononuclear (M-MDSCs) subsets, facilitate tumor immune evasion through the production of pro-inflammatory cytokines (e.g., IL-6, TGFβ, CCL2, IL-10) and immunosuppressive factors (e.g., iNOS, NO, Arg1, and ROS) (13, 14). Recent research has begun to elucidate the complex interactions between MDSCs, immune evasion, and chemoresistance; however, the specific mechanisms underlying these processes remain ripe for further investigation (15–18). Further, increased MDSCs infiltration is associated with dysregulation of the Notch signaling pathway, which is known to contribute to cancer progression (19–21).

Notch signaling involves receptor-ligand interactions that trigger proteolytic cleavage of Notch receptors, promoting the nuclear translocation of the notch intracellular domain (NICD) to regulate gene transcription, including hairy and enhancer of split-1 (HES1) linked to tumor progression (19, 20, 22–25). The stability and function of NICD are influenced by glycogen synthase kinase 3β (GSK3β), a kinase that plays a key role in cell proliferation, differentiation, and survival (26). It is constitutively active in resting cells but can be inactivated through serine 9 phosphorylation in pathways that include PI3K/Akt and SGK1, among other kinases, which are aberrantly activated in tumor cells (26–30). Its role in the Notch pathway is context-dependent, as GSK3β can affect transcription and protein stability (26), thereby influencing progression. Interestingly, it has been suggested that stress hormones can enhance cancer cell viability and invasion in a Notch-dependent manner (31).

This study aimed to establish the relationship between chronic stress, MDSCs biology, and Notch signaling to understand how these factors support an immunosuppressive tumor microenvironment and drive OC progression. We show that chronic restraint stress significantly induced OC progression by promoting the infiltration of MDSCs into the TME. We demonstrated that stress hormones, particularly corticosterone, led to the enrichment of PMN-MDSCs, decreased M-MDSC populations, activated the Notch signaling pathway, and enhanced the suppressive activity, all of which potentially support the immunosuppressive functions of MDSCs. Additionally, we found that corticosterone inhibits GSK3β activity, which led to the upregulation of key Notch signaling pathway members (NICD and HES1) in OC cells, thus facilitating tumor progression and metastasis. These findings highlight the significance of addressing the tumor-promoting effects of chronic stress in patients with OC and provide a compelling justification for developing behavioral interventions and therapeutic strategies to mitigate these effects, ultimately enhancing patient outcomes.

## 2 Materials and methods

### 2.1 Cell lines and culture conditions

The ID8 cell line was purchased from Ubigene (Cat#YC-C103-Luc-P), and the IG10 cell line was obtained from Katy Roby at the University of Kansas (Lawrence, Kansas). Both cell lines were cultured in RPMI 1640 medium supplemented with 1% antibiotic/antimycotic and 10% fetal bovine serum. All cell lines were grown in 95% O_2_ and 5% CO_2_ at 37°C. All experiments were conducted with cultures at 70–80% confluence. After reaching around 70% cell density, the cells were trypsinized, seeded into 6-well plates, and maintained under the same conditions. Both cell lines were stimulated in these plates with 10 μM of norepinephrine (NE) (Sigma, Cat#N5785) in dH_2_O, 10 μM of epinephrine (EPI) (Sigma, Cat#E4375) in dH_2_O, or 10 μM of corticosterone (CC) (Sigma, Cat#27840) in ethanol in serum-free media for 30 minutes, 1 hour, 12 hours, 24 hours, and 48 hours, depending on the experiment. For the inhibition experiments, IG10 cells were treated and incubated with 20 μM of N-[N-(3,5-Difluorophenacetyl)-L-alany]-S-phenylglycine t-butyl ester (DAPT) (Sigma, Cat#D5942) and 10 μM Mifepristone (RU-486) (Sigma, Cat#M8046) for 1 hour before adding 10 μM of corticosterone, which was incubated for another hour in serum-free media, preceding the protein extraction protocol for Western blot analysis.

### 2.2 Animal experiments

For animal studies, 8- to 12-week-old C57BL/6 female mice were obtained from Taconic Laboratories and housed at the Ponce Research Institute Animal Facility. All experiments received approval from the Institutional Animal Care and Use Committee at Ponce Health Sciences University. The investigator handled all mice one week prior to the start of each experiment. Mice were inoculated intraperitoneally with either 1 x 10^^6^ ID8^Luc^ or IG10^Luc^ murine ovarian cancer cell lines. Following the inoculation, mice were monitored weekly for tumor growth (IG10^Luc^ for four weeks; ID8^Luc^ for two weeks) using the Revvity IVIS Lumina S5 (Waltham, MA) through the intraperitoneal injection of Luciferin (Revvity, Cat#122799) at a dosage of 10 µL/g of body weight, with each mouse receiving 150 mg Luciferin per kg body weight, as per the manufacturer’s protocol. The Luciferin injection was performed after shaving the mice in the intraperitoneal area and 10–15 minutes before *in vivo* imaging. Once tumor growth was confirmed *via* imaging, mice were randomized into the stress (N = 20) and control groups (N = 20). To induce chronic stress in mice, we utilized a daily physical-restraint stress model that restricted their movement for two hours each day until the experiment concluded (32, 33). Access to food and water was restricted for all groups during the chronic restraint stress protocol. Five mice from each group (stress and control) were sacrificed weekly over four weeks. Tumor burden was assessed by examining the peritoneal cavity, removing any tumor nodules present, and calculating the combined tumor weight per mouse. Tumor samples were divided for flow cytometry assays, formalin fixation, and mounting in paraffin blocks for immunohistochemistry and immunofluorescence analysis. Bone marrow was extracted from the femurs and processed for flow cytometry assays. Additionally, blood was processed for serum and stored at −80°C for ELISA.

### 2.3 Quantitative real-time PCR

To investigate the effects of stress hormones on the Notch signaling pathway as well as β-adrenergic and glucocorticoid receptor mRNA expression, total RNA was extracted using TRIzol Reagent, following the manufacturer’s instructions (Life Technologies, Cat#15596018). RNA was quantified and reverse transcribed (Bio-Rad, Cat#170-8891), and quantitative real-time PCR (qPCR) analysis was performed (Bio-Rad, Cat#170-8882). The mRNA levels of *Notch1, Notch2, Notch3, Jagged1, Jagged2, Hes1, Adrb1* (QIAGEN, Cat#PPM05035A-200), *Adrb2* (QIAGEN, Cat#PPM04265C-200), and *GR* were evaluated; refer to **Supplementary Table 2** for primer sequences. *Gapdh* mRNA levels were used as a control. The 2−ΔΔCT method was employed to calculate fold changes relative to the control, and the average of three independent experiments was reported.

### 2.4 Western blotting

Cells were detached and lysed from 6-well plates using scrapes; then, proteins were fractionated into cytosolic and nuclear components following the manufacturer’s protocol from the Nuclear/Cytosol Fractionation Kit (Abcam, Cat# ab289882). Protein concentration was determined using the BCA Protein Assay Kit (Thermo Scientific, Cat#23225). An equal amount of protein (30 µg) was analyzed by SDS-PAGE (Bio-Rad, Cat#1610173) and transferred to a polyvinylidene difluoride membrane (PVDF) (Bio-Rad, Cat#1620177) using the Trans-Blot Turbo Transfer Kit (Bio-Rad, Cat#1704275). Membranes were blocked with 5% Bovine Serum Albumin (BSA) (Sigma, Cat#A9647) in Tris-Buffered Saline Tween (TBS-T) at room temperature for 1 hour and incubated overnight at 4°C with primary antibodies (**Supplementary Table 3**). Then, blots were washed three times for 5 minutes each with TBS-T and incubated with secondary antibodies (**Supplementary Table 3**) for 1 hour at room temperature. Blots were detected using Clarity Western ECL Substrate (Bio-Rad, Cat#170-5061) with a ChemiDoc XRS+ Imaging System (Bio-Rad). The average of three independent experiments was reported. Western blots were quantified using ImageJ software (Bethesda, MD, USA). Band densities were normalized to β-actin or Histone H3 from the same sample and blot to control for loading. All western blot data are presented as protein expression compared to the control.

### 2.5 Protein-protein interaction network

The Search Tool for the Retrieval of Interacting Genes/Proteins (STRING; string-db.org) database provides associations and links between query genes and proteins along with a protein-protein interaction (PPI) network. The presented network, along with the gene enrichment analysis of differentially expressed cytokines identified earlier, was generated using the STRING-free online platform (version 12.0). Key network properties include edges, which represent the number of interactions; nodes, indicating the total proteins within the network; node degree, reflecting the average interactions; and the clustering coefficient, which measures the likelihood of cluster formation within the network. A clustering coefficient close to 1 suggests a higher probability of cluster formation, while the PPI enrichment p-value signifies statistical significance.

### 2.6 Enzyme-linked immunosorbent assay

Serum samples from tumor-bearing mice that were subjected to restraint stress were used to measure corticosterone levels *via* ELISA, utilizing the Mouse Corticosterone ELISA kit (R&D Systems, #KGE009) according to the manufacturer’s protocol. Samples were analyzed in technical duplicates, and the data reflect the average concentration for each sample.

### 2.7 Cytokine multiplex assay and analyses

Individual cytokine levels of serum samples from tumor-bearing mice were quantified using a murine 32 pro-inflammatory cytokine/chemokine panel (Millipore Sigma, Cat#MCYTOMAG-70K-PX32). Cytokine panels were used according to the manufacturer’s established protocols. Statistical analyses of these data were conducted as follows. First, cytokines with measurements below the lower limit of detection in at least 30% of samples were categorized as either present or absent (LIF, GM-CSF, MIP-1α, M-CSF, IL-7, IL-12 p70, IL-12 p40, IL-4, IFN-γ, IL-3, VEGF). The remaining twenty cytokines (IP-10, KC, IL-9, IL-1β, LIX, G-CSF, MIP-1β, MCP-1, MIG, IL-15, IL-10, IL-1α, IL-17, IL-5, IL-6, IL-2, MIP-2, RANTES, TNF-α, Eotaxin) were used for further analysis using the tidyverse package (34) in R Statistical Software (35). Heatmaps were created using the log-transformed cytokine levels of the stressed and control groups using the R package pheatmap (36). To reduce the dimensionality of the cytokine concentration data and identify the factors most contributing to differences between the stress and control groups, principal component analysis was performed on the log-transformed cytokine levels using data from both groups. The R packages factoextra (37) and ggfortify (38, 39) packages were used to construct a biplot along dimensions 1 and 3 of the PCA results.

### 2.8 Immunohistochemistry

Formalin-fixed, paraffin-embedded tumor tissues were utilized to identify Notch1, Jagged2, HES1, NICD, ADRB2, GR, pS9-GSK3β, F4/80^+^, CD4^+^ and CD8^+^ expression through IHC analysis (**Supplementary Table 3**). First, slides were heated at 60°C and then deparaffinized using an Xylene substitute. To inhibit endogenous peroxidase activity, we incubated the slides with 3% hydrogen peroxide for 15 minutes at room temperature in the absence of light. The antigen retrieval step was conducted at 95°C using TRIS-EDTA buffer at a pH of 9.0 (Notch1, Jagged2, and ADRB2) and Citrate at pH 6.2 (NICD, HES1, GR, pS9-GSK3β, F4/80^+^, CD4^+^ and CD8^+^) for 40 minutes, followed by a 20-minute cool-down period. Protein Block solution (Abcam, Cat#ab64226) was added to the tissues and incubated for 1 hour at room temperature. Primary antibodies were applied at the above dilutions and incubated overnight at 4°C in a humidity chamber. We used the Multilink & Streptavidin Detection Systems (BioGenex, Cat#LP000-ULE) as secondary antibodies. Diaminobenzidine (DAB) (BioGenex, Cat#HK542-XAKE) was applied for 1–10 minutes while constantly monitoring under a brightfield microscope. Slides were then dipped in hematoxylin for counterstaining and immediately washed with tap water for 5 minutes. After drying, coverslips were mounted with a cytoseal medium (Thermo Scientific, Cat#8310-16). Four representative high-power fields (HPF) were captured for each tumor section, and the images were further analyzed using ImageJ software (Bethesda, MD, USA).

### 2.9 Immunofluorescence

Formalin-fixed, paraffin-embedded tumor tissues were analyzed for MDSCs infiltration using immunofluorescence staining. Sections were then washed three times in PBS for 10 minutes each, followed by a 1-hour incubation in Protein Block solution (Abcam, Cat#ab64226). The tissues were incubated overnight with primary antibodies CD11b and Gr-1 (**Supplementary Table 3**). A tissue section served as a negative control with PBS instead of a primary antibody. On the second day, the tissues were washed three times for 5 minutes in PBS, followed by a one-hour incubation with Alexa Fluor 488 Goat anti-Rat secondary antibody and Alexa Fluor Goat anti-Rabbit secondary antibody. After washing three times with PBS for 5 minutes each, sections were incubated for 5 minutes in NucBlue Fixed Cell Stain Ready Probes (DAPI) (Invitrogen, Cat#R37606). Tissues were washed three times for 5 minutes each with PBS buffer and then mounted on slides using ProLong Gold antifade reagent (Invitrogen, Cat#P36934). Four representative high-power fields were captured for each tumor section, and the images were further analyzed using ImageJ software (Bethesda, MD, USA).

### 2.10 Immunocytochemistry

After the treatment and incubation with the stress hormones described above, cells were washed twice with PBS and fixed using 4% paraformaldehyde (PFA) for 20 minutes at 4°C. After fixation, the cells were permeabilized with 0.5% Triton for 20 minutes at room temperature (RT). The cells were blocked with 2% BSA for 1 hour at RT. Following blocking, the cells were washed twice with PBS and then incubated with the NICD antibody (Cell Signaling, Cat#4147S) for 3 hours at RT. Next, the cells were washed twice with PBS and incubated with the secondary antibody Alexa Fluor 488-Conjugated AffiniPure F’(ab’) Fragment Goat Anti-Rabbit IgG (Jackson ImmunoResearch, Cat#111-546-047) for 1 hour at RT. Subsequently, the cells were washed twice with PBS and incubated with DAPI for 5 minutes at RT. Finally, the cells were washed twice with PBS, dried, and then mounted on slides using ProLong Gold Antifade reagent. Four representative high-power fields were captured for each tumor section, and the images were further analyzed using ImageJ software. (Bethesda, MD, USA).

### 2.11 Development of mouse MDSC from bone marrow precursors

Bone marrow cells were isolated from the femurs of C57BL/6 mice under sterile conditions. Briefly, femurs were aseptically dissected, and both ends were cut to expose the marrow cavity. The bone marrow was flushed with phosphate-buffered saline (PBS) containing 2% fetal bovine serum (FBS) using a 25G needle. Cell suspensions were filtered through a 70-µm cell strainer to remove debris and centrifuged at 300 × g for 5 min. Red blood cells were lysed with RBC lysis buffer for 5 minutes at 4°C, followed by washing and resuspension in complete RPMI-1640 medium supplemented with 10% FBS and 1% antibiotic/antimycotic solution. Bone marrow-derived cells (BM-MDSCs) were seeded at a density of 1 × 10^6^ cells/mL in complete RPMI medium containing 10 ng/mL granulocyte-macrophage colony-stimulating factor (GM-CSF) and 10 ng/mL granulocyte colony-stimulating factor (G-CSF). Cells were incubated for 4 days at 37°C in a humidified 5% CO_2_ atmosphere to induce MDSCs differentiation. On day 4, differentiated myeloid cells were collected for subsequent experiments.

### 2.12 T-cells isolation

Splenic CD8^+^ T cells were isolated from naïve C57BL/6 mice. Spleens were mechanically dissociated through a 70-µm strainer into cold PBS supplemented with 2% FBS. Red blood cells were lysed with RBC lysis buffer for 5 minutes at 4°C, and CD8^+^ T cells were purified by magnetic bead separation using a CD8a^+^ T Cell Isolation Kit (Miltenyi Biotec, Cat#130-117-044) following the manufacturer’s instructions.

### 2.13 *In vitro* and *ex vivo* T-cell suppression assays

To evaluate the effects of stress signaling on MDSCs suppression, murine BM-MDSCs were plated in 6-well plates and treated with 10µM NE, 10µM EPI, or 10µM CC for 24 hours. Following hormone exposure, murine BM-MDSC were co-cultured with CD8^+^ T cells at varying MDSC:T-cell ratios (1:1 to 1:32) in round-bottom 96-well plates bound with anti-CD3 and anti-CD28 (1 μg/ml each) (Miltenyi Biotec, Cat#130-093-627) for 3 days. T-cell activation/suppression was assessed with Granzyme B and IFN-γ by flow cytometry.

### 2.14 Flow cytometry assay

Bone marrow and tumor samples (60 mg) were isolated from tumor-bearing mice subjected to restraint stress and processed for FACS analysis. Bone marrow samples were centrifuged at 4°C for 10 minutes at 1,200 rpm. The cells were then washed with 1 mL of Red Blood Cell Lysis Buffer (Roche, Cat#11814389001) before being centrifuged again for another 10 minutes at 4°C at 1,200 rpm. Supernatants were discarded, and the cells were incubated with primary antibodies for CD11b-APC, Ly-6G-PE/Cy7, and Ly-6C-FITC for 1 hour at 4°C (**Supplementary Table 3**). After incubation, samples were centrifuged at 4°C and 1,200 rpm for 7 minutes, and the pellet was collected and resuspended in 800 μL of 0.5% PFA. For tumor samples, we followed an established laboratory protocol (32). Cells were acquired using a FACS Melody (BD Biosciences), analyzed through density plots, and populations of CD11b^+^/Ly-6G^+^ and CD11b^+^/Ly-6C^+^ were selected. FlowJo and Floreada.io were used to analyze the histograms and plots.

### 2.15 Statistical analyses

Protein expression and cell quantification (MDSCs) were evaluated using ImageJ software and analyzed with GraphPad Prism (version 9.4.1). T-tests or Mann-Whitney tests were conducted for parametric and non-parametric values, respectively, as determined by Shapiro-Wilk normality tests. Ordinary One-way ANOVA or Two-way ANOVA with Tukey’s multiple comparison test was used to compare more than two groups. Experiments were conducted independently at least three times. Data are reported as mean ± SEM. All results are presented as two-tailed p-values, with *p* < 0.05 considered statistically significant.

## 3 Results

### 3.1 Restraint stress promotes the infiltration of MDSCs, accelerates tumor progression, and increases corticosterone levels in OC-tumor-bearing mice

**Figure 1:**
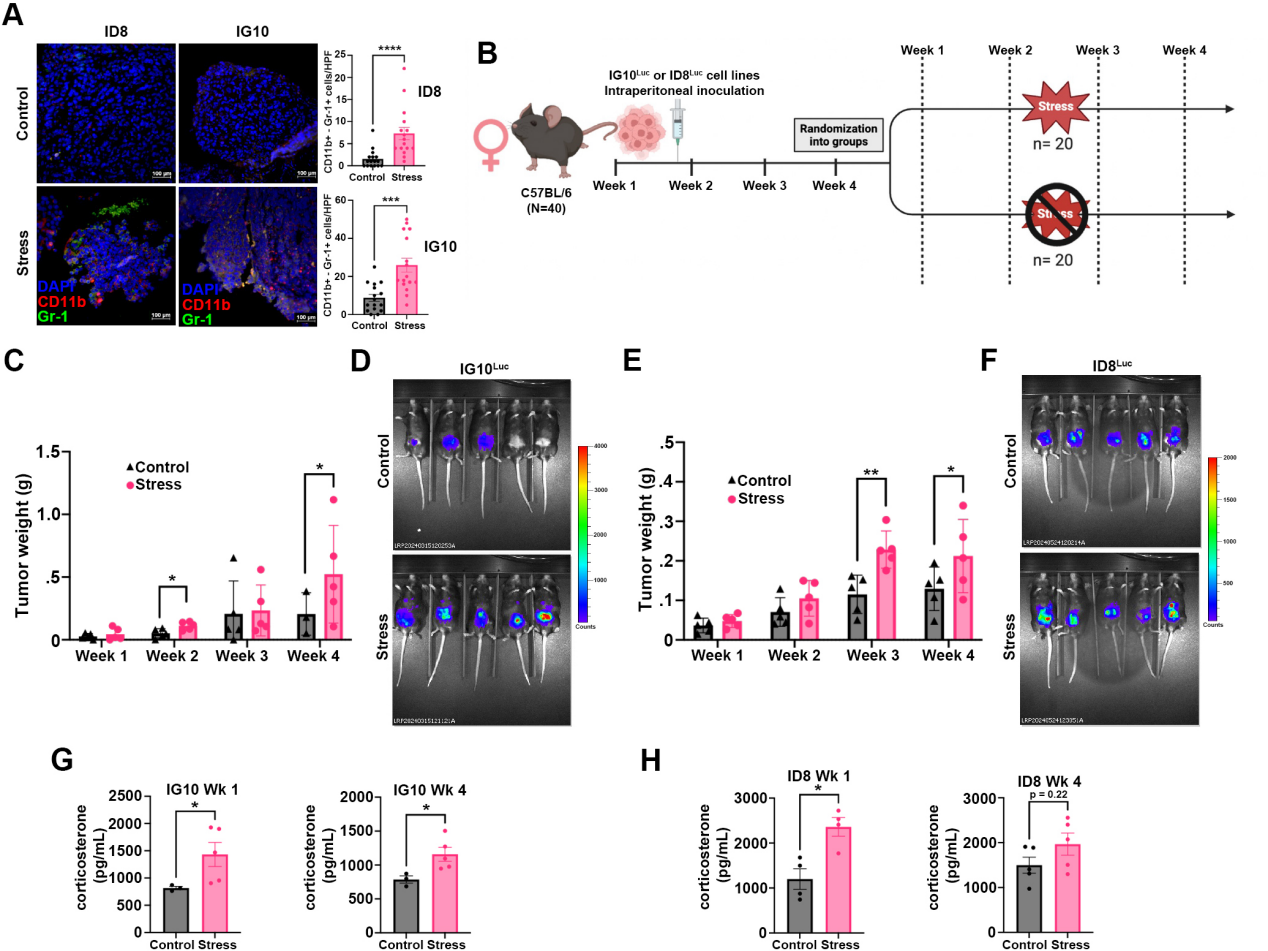
Restraint stress promotes MDSCs infiltration, accelerates tumor growth and increase corticosterone levels in OC-bearing mice. **(A)** Representative images (40X) and quantification of MDSCs CD11b^+^/Gr-1^+^ cells from ID8 (N = 4) and IG10 (N = 4) tumor samples from mice subjected to restraint stress. **(B)** Experimental *in vivo* design of daily restraint stress of ID8^Luc^ and IG10^Luc^ tumor-bearing mice, created in BioRender.com. Tumor weight measured during necropsy and tumor monitoring by *in vivo* luminescence imaging at 8 weeks after injection of **(C, D)** IG10^Luc^ and **(E, F)** ID8^Luc^ OC cells (N = 5; per each group-week). Luminescence scale represented by heat map where cooler colors indicate less tumor growth and warmer colors increase tumor growth. **(G)** Corticosterone levels (pg/mL) in serum samples in IG10^Luc^ mice (Control, N = 3; Stress, N = 5) and **(H)** ID8^Luc^ mice (Control, N = 4-5; Stress, N = 4-5). Statistical analysis was performed using the Mann-Whitney test and Unpaired T-test. Data presented are represented as mean ± SEM (*p<0.05, **p < 0.01, ***p < 0.001, ****p < 0.0001).

Previous studies conducted in our laboratory demonstrated that chronic stress contributes to OC TME inflammation and immunosuppression (32, 33). However, the role of MDSCs in these processes remains unclear. To evaluate whether chronic stress induces MDSCs expansion in the OC TME, we analyzed tumor samples from two well-characterized syngeneic mouse models for OC, ID8 and IG10, subjected to daily restraint stress versus controls through immunofluorescence (IF) staining. A substantial increase in CD11b^+^/Gr-1^+^ cells within the tumor was detected in mice subjected to chronic daily restraint stress (**Figure 1A**). Quantitative analysis confirmed this finding, demonstrating elevated infiltration of CD11b^+^/Gr-1^+^ cells in both syngeneic mouse models, ID8 (*p* < 0.0001) and IG10 (*p* = 0.0001) (**Figure 1A**) after restraint stress. These findings indicate a role for chronic stress in OC-associated MDSCs biology.

To characterize the effects of chronic restraint stress on OC progression and its biological significance, we conducted longitudinal *in vivo* studies using ID8^Luc^ and IG10^Luc^ inoculated mice subjected to chronic daily restraint stress or control (**Figure 1B**). Mice subjected to daily restraint stress exhibited a significant increase in tumor weight in both syngeneic mouse models (IG10^Luc^ and ID8^Luc^) (**Figures 1C–F**). This effect was evident throughout the experimental period, with a particularly pronounced increase in tumor growth during the later weeks of the experiment (weeks 3-4). Furthermore, corticosterone levels were significantly elevated in the serum of stressed tumor-bearing mice after one week of restraint stress [ID8^Luc^ (*p* = 0.0286) and IG10^Luc^ (*p* = 0.0357)] (**Figures 1G, H**). Interestingly, after four weeks of restraint stress, corticosterone levels were elevated in IG10^Luc^-bearing mice (p=0.0357), but not significantly in ID8^Luc^-bearing stressed mice (*p* = 0.2222**) (****Figures 1G, H**). These findings support that chronic restraint stress can significantly accelerate ovarian cancer progression in syngeneic mouse models, leading to an increased tumor burden and metastasis.

### 3.2 Restraint stress-induced enrichment and polarization of MDSCs in the bone marrow and tumors of OC-tumor-bearing mice

**Figure 2:**
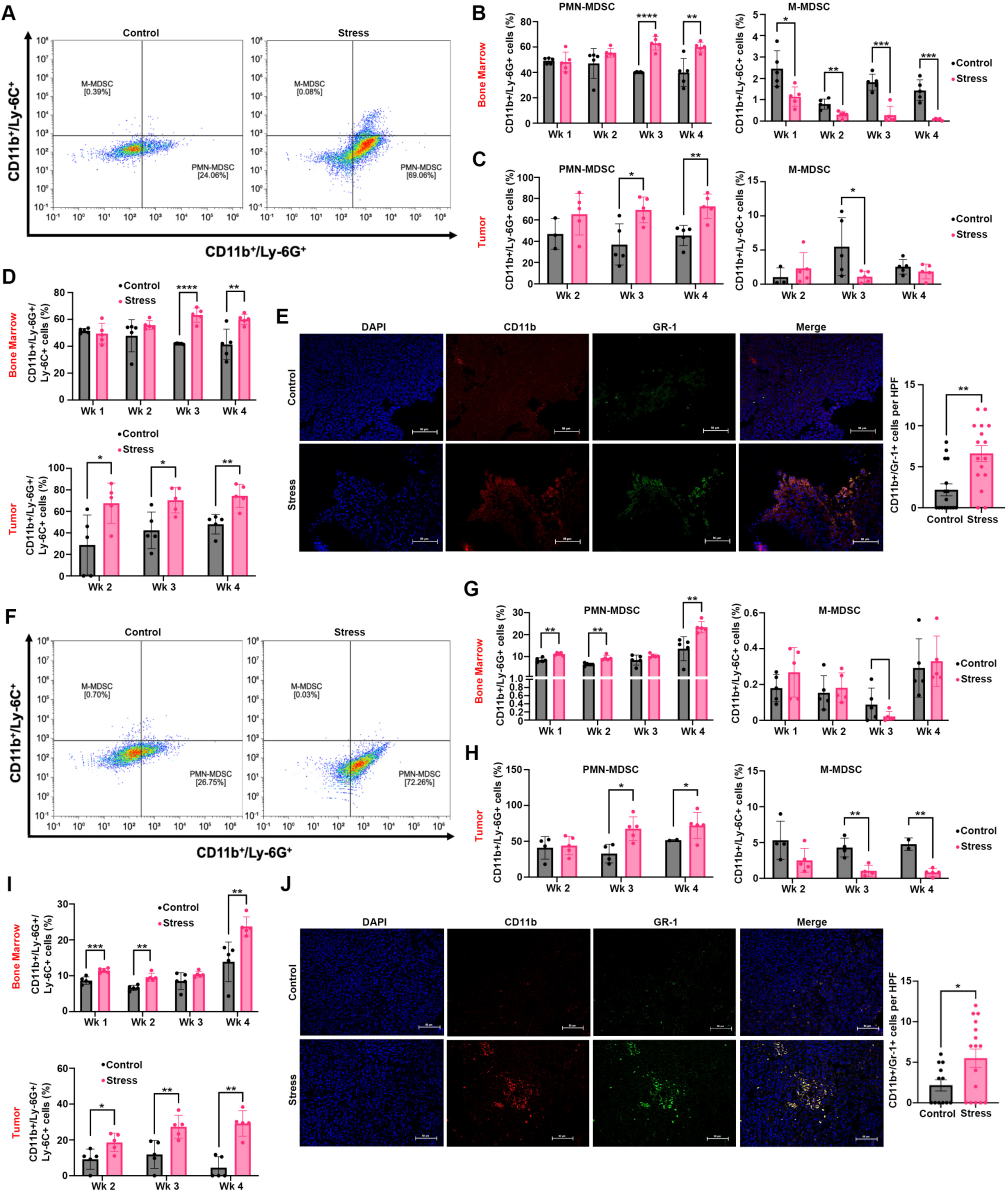
Restraint stress induces MDSCs enrichment and polarization in the bone marrow and tumors of OC-bearing mice. **(A)** Cellular expression of polymorphonuclear (PMN)-MDSCs (CD11b^+^/Ly-6G^+^) and mononuclear (M)-MDSCs (CD11b^+^/Ly-6C^+^) in tumor samples from ID8^Luc^ stressed and control mice at week 4. **(B, C)** MDSCs populations in the bone marrow and tumor samples from ID8^Luc^ mice. **(D)** Total MDSC populations in the bone marrow and tumor samples from ID8^Luc^**(E)** Representative images (40X) and quantification of MDSCs CD11b^+^/Gr-1^+^ cells from ID8^Luc^ (N = 4). **(F)** Cellular expression of PMN-MDSCs and M-MDSCs in tumor samples from IG10^Luc^ stressed and control mice at week 4. **(G, H)** MDSCs populations in the bone marrow and tumor samples from IG10^Luc^ mice. **(I)** Total MDSCs populations in the bone marrow and tumor samples from IG10^Luc^**(J)** Representative images (40X) and quantification of MDSCs CD11b^+^/Gr-1^+^ cells from IG10^Luc^ (N = 3-4). Statistical analysis was performed using the Mann-Whitney test and Unpaired T-test. Data presented are represented as mean ± SEM (*p < 0.05; **p < 0.01; ***p < 0.001; ****p < 0.0001). (N = 40; 20 mice per model (ID8^Luc^ and IG10^Luc^).

We collected bone marrow and tumors from the longitudinal study to test whether chronic stress induces MDSCs expansion in the bone marrow and OC tumors by flow cytometry assays. Our results show that daily restraint stress increases PMN-MDSCs (CD11b^+^/Ly-6G^+^) frequency and decreases M-MDSCs (CD11b^+^/Ly-6C^+^) in the bone marrow and tumors of the ID8^Luc^ model, with a notable increase in PMN-MDSCs during the later weeks of the experiment (weeks 3-4), corresponding to the tumor burden (**Supplementary Figure S1A-C**; **Figures 2A–C**). Additionally, restraint stress increased total MDSCs in the tumors and bone marrow of the ID8^Luc^ model (**Figure 2D**). Furthermore, we assessed MDSCs infiltration in paraffin-embedded tumor tissue. Our data revealed a significant increase in MDSCs in tumors from mice subjected to daily restraint stress (ID8^Luc^: *p* = 0.0019) (**Figure 2E**). Similar results were found in the IG10^Luc^ syngeneic mouse model, leading to an increase of PMN-MDSCs and a decrease of M-MDSCs in the bone marrow and tumors of mice subjected to restraint stress, specifically in weeks 3 and 4 (**Figures 2F–I**). Moreover, restraint stress led to a significant increase in total MDSCs infiltration in tumors from the IG10^Luc^ model (*p* = 0.0479) (**Figure 2J**). These findings suggest a direct association between chronic restraint stress and MDSCs enrichment in the bone marrow and tumors of OC-bearing mice.

### 3.3 Restraint stress alters serum cytokine profiles while promoting macrophage accumulation and decreasing T cell populations in the TME of OC-bearing mice

**Figure 3:**
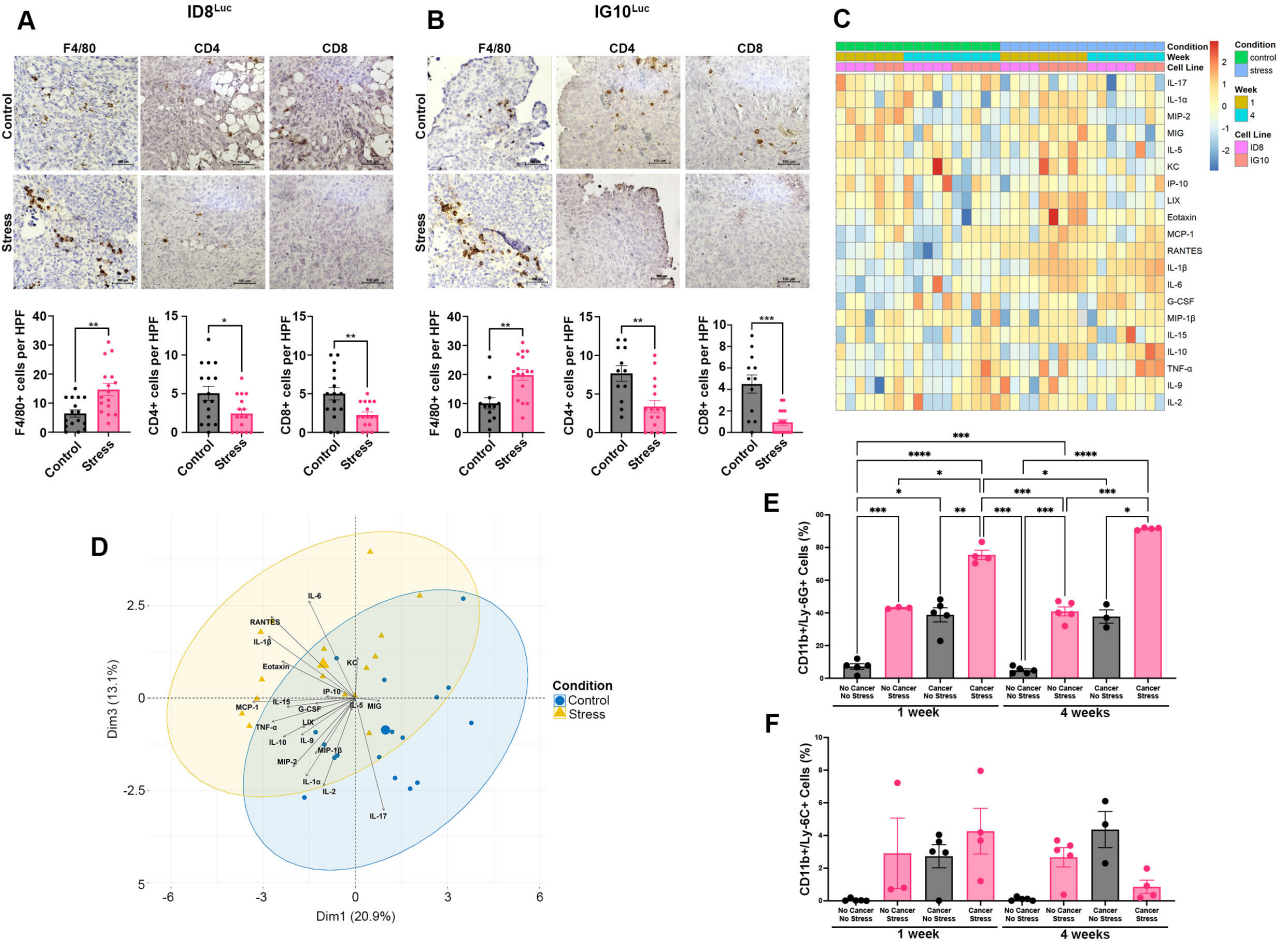
Restraint stress alters serum cytokine profiles and leads to macrophage accumulation and decreases T cell populations in the TME of OC-bearing mice. **(A, B)** Representative immunohistochemistry (IHC) images (40X) and quantification for F4/80^+^ macrophages, CD4^+^ and CD8^+^ T cell subtypes in tumors from ovarian tumor-bearing mice (ID8^Luc^, N = 4; IG10^Luc^, N = 3-4) subjected to restraint stress for four weeks. **(C)** Heatmap and **(D)** Biplot of PCA dimensions 1 and 3 for measurable cytokine levels in the serum of stressed and control ovarian tumor-bearing mice (ID8^Luc^ and IG10^Luc^). **(E)** PMN-MDSCs and **(F)** M-MDSCs populations in the bone marrow from IG10^Luc^ mice (Total N = 34). Statistical analysis was performed using the Mann-Whitney test or One-way ANOVA (Brown-Forsythe and Welch ANOVA test) with Dunnett’s T3 multiple comparison. Data presented are represented as mean ± SEM. (*p < 0.05; **p < 0.01; ***p < 0.001; ****p < 0.0001).

To investigate whether chronic restraint stress could influence immune cell populations in the ovarian tumor microenvironment, we examined F4/80^+^ macrophages and CD4^+^ and CD8^+^ T-cell subtypes using immunohistochemistry (IHC) on paraffin-embedded tumor samples from ID8^Luc^ and IG10^Luc^ tumor-bearing mice that underwent daily restraint stress versus control for four weeks. Our results indicated a higher accumulation of F4/80^+^ macrophages in tumors from mice exposed to restraint stress (ID8^Luc^: *p* = 0.0039; IG10^Luc^: *p* = 0.0011) (**Figures 3A, B**). Conversely, there was a noted decrease in the infiltration of CD4^+^ (ID8^Luc^: *p* = 0.0349; IG10^Luc^: *p* = 0.0031) and CD8^+^ (ID8^Luc^: *p* = 0.0071; IG10^Luc^: *p* = 0.0008) T cells in both syngeneic mouse models. These data suggest that daily restraint stress enhances an immunosuppressive ovarian tumor microenvironment.

To characterize the effect of restraint stress on cytokine profiles in ID8^Luc^ and IG10^Luc^ OC-bearing mice, we performed a cytokine multiplex assay on blood serum from the *in vivo* experiments. The data obtained from these analyses are shown in **Supplementary Table 1**. To further visualize the changes seen between groups, we created a heatmap and analyzed the data using principal component analysis. The heatmap displays serum cytokine levels from control and stressed mice at two time points (weeks 1 and 4) (**Figure 3C**). Distinct clustering patterns were observed between stressed and control mice, indicating that stress induces a broad shift in cytokine expression. Several pro-inflammatory cytokines, including IL-6, IL-1β, MCP-1 (CCL-2), RANTES, TNF-α, and eotaxin, were elevated at week 4 under stress conditions, while others, such as IL-2 and IL-17, were reduced. Moreover, in the principal component analysis (PCA), stressed (yellow) and control (blue) mice are separated along the first principal component (Dim1), which explains 20.9% of the variance. (**Figure 3D**). The cytokines most strongly associated with this separation include RANTES, IL-6, MCP-1 (CCL-2), IL-1β, TNF-α, and eotaxin, which are linked to the stress groups. Overall, these results demonstrate that chronic restraint stress alters the systemic cytokine milieu in OC-bearing mice, inducing inflammatory and chemotactic factors that may promote immunosuppression and disease progression, while simultaneously suppressing cytokines that support adaptive immune responses.

Given that the interaction between factors modulated by chronic stress and cancer is bidirectional, and considering that both tumors and chronic stress influence the frequency of MDSCs, an *in vivo* experiment was conducted utilizing OC-bearing mice (IG10^Luc^) and non-OC-bearing mice, with a group subjected to chronic daily restraint stress (**Supplementary Figure S2A**). Flow cytometry analysis of bone marrow from these mice showed that both chronic stress and tumor-bearing conditions independently increased the frequency of PMN-MDSCs compared to the mice with no tumor and not subjected to stress (week 1; *p* = 0.0003; *p* = 0.0141 and week 4; *p* = 0.0008; p=0.0848, respectively) (**Figure 3E**; **Supplementary Figure S2B**). Furthermore, the combination of both chronic stress and tumor burden further amplified this effect, demonstrating the highest frequency of PMN-MDSCs compared to all other groups (cancer/no stress (p=0.0035; p=0.0035), no cancer/stress (*p* = 0.0118; *p* = 0.0006), and no cancer/no stress (*p* < 0.0001; *p* < 0.0001)) after one or four weeks of daily restraint stress, respectively. On the other hand, we did not observe a significant difference among groups in M-MDSCs populations after one or four weeks of daily restraint stress (**Figure 3F**; **Supplementary Figure S2B**). These findings suggest that both chronic stress and tumors promote the expansion of PMN-MDSCs in the bone marrow of OC-bearing mice subjected to daily restraint stress. These data support our previous observations and suggest a possible mechanism by which chronic stress may enhance tumor-induced immunosuppression.

### 3.4 Stress hormones promote PMN-MDSCs enrichment, M-MDSCs decline, and augment the suppressive activity of MDSCs on T-cells

**Figure 4:**
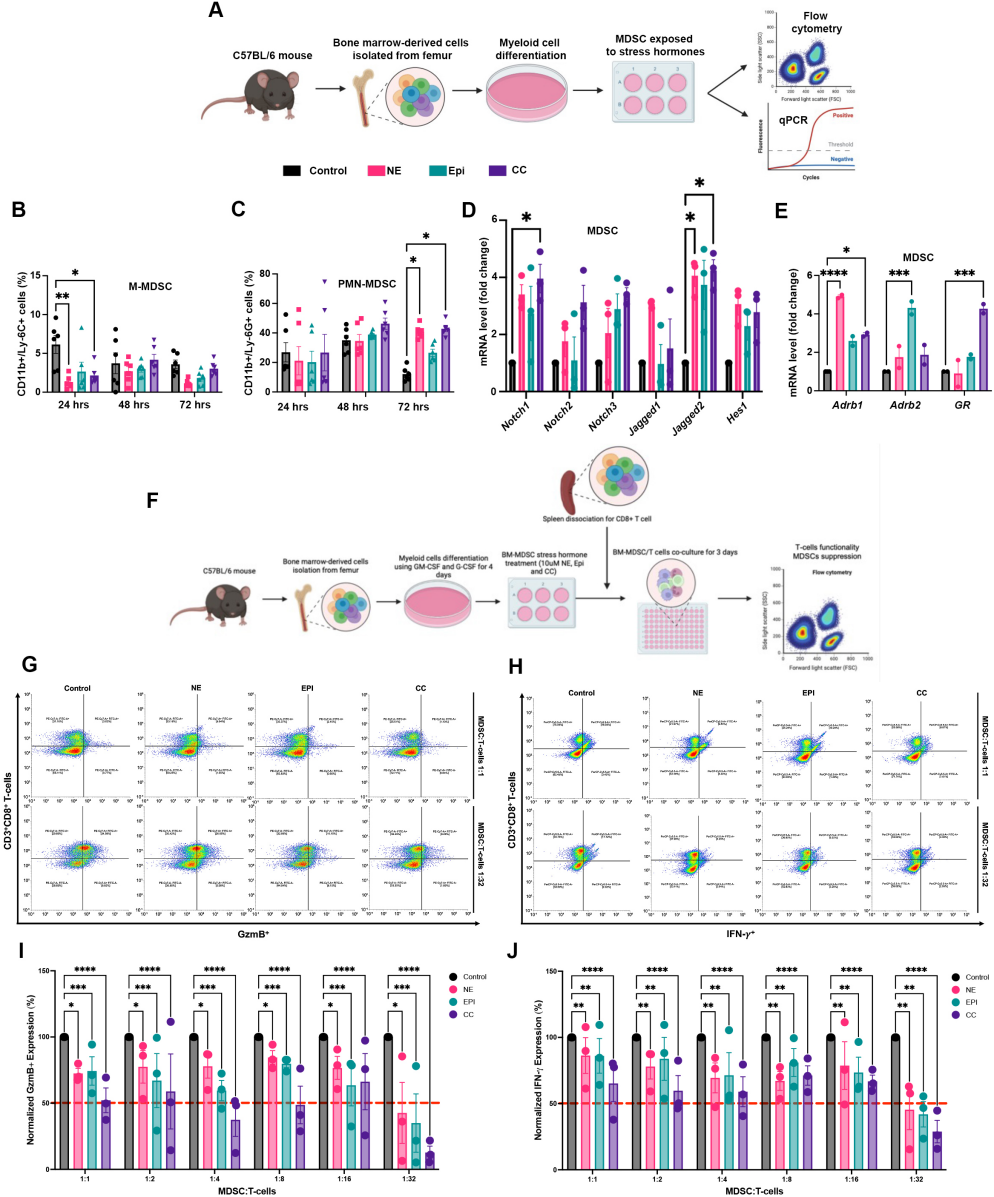
Stress hormones promote PMN-MDSCs enrichment, M-MDSCs decline, and augment the suppressive activity of MDSCs on T-cells. **(A)** Experimental design for MDSCs *ex-vivo* generation and stress hormones treatment, created in BioRender.com. **(B)** Quantification of cellular expression of CD11b^+^/Ly-6C^+^ (M-MDSCs) and **(C)** CD11b^+^/Ly-6G^+^ (PMN-MDSCs) cells measured by flow cytometry on C57BL/6 bone marrow-derived MDSC treated with stress hormones for 72 hrs. **(D)** Notch signaling pathway **(E)** β-adrenergic and glucocorticoid receptor mRNA levels after stress hormones treatment at 72 hrs measured using qPCR. **(F)** Experimental design for MDSCs *ex-vivo* generation, stress hormones treatment, and co-culture with CD8+ T-cells, created in BioRender.com. Representative cellular expression of GzmB+ **(G)** and IFN-γ+ **(H)** in CD8+ T-cells co-cultured with MDSCs at different ratios (1:1 and 1:32). Normalized quantification of cellular expression of GzmB+ **(I)** and IFN-γ+ **(J)** in CD8+ T-cells co-cultured with MDSCs at different ratios measured by flow cytometry. Statistical analysis was performed using Two-way ANOVA with Tukey multiple comparison correction. Data presented are represented as mean ± SEM of three independent experiments (n=3) (*p < 0.05; **p < 0.01; ***p < 0.001; ****p < 0001).

To characterize the impact of stress hormones on MDSC biology, we isolated bone marrow from C57BL/6 mice and generated MDSCs *ex vivo* (40) (**Figure 4A**). After differentiating with GM-CSF (10 ng/mL) and G-CSF (10 ng/mL) for 96 hours, MDSCs were treated with stress hormones (10 μM NE, 10 μM EPI, or 10 μM CC) for 72 hours. MDSC subsets were evaluated by flow cytometry. Additionally, RNA was extracted to measure the mRNA levels of the Notch signaling pathway members, β-adrenergic receptors, and glucocorticoid receptors to assess how stress hormones modulate this pathway in MDSCs. Results showed decreased M-MDSCs (CD11b^+^/Ly-6C^+^) expansion at 72 hours in the stress-hormone-treated groups (**Figure 4B**), while PMN-MDSCs (CD11b^+^/Ly-6G^+^) were sustained and enriched in the stress hormone-treated groups, particularly with NE (*p* = 0.0391) and CC (*p* = 0.0194); no significant changes were observed with EPI (*p* = 0.8336) (**Figure 4C**). Conversely, CC elevated the mRNA levels of *Notch1* (*p* = 0.0455) and *Jagged2* (*p* = 0.0160). Additionally, mRNA levels of *Adrb1* (*p* = 0.0142) and *GR* (*p* = 0.0003) were induced as well (**Figures 4D, E**). Furthermore, NE increased the levels of *Jagged2* (*p* = 0.0316) and *Adrb1* (*p* < 0.0001), while EPI induced the mRNA levels of *Adrb2* (*p* = 0.0002). These results suggest that stress hormones may influence MDSCs subset development, driving polarization and differentiation. Notably, CC activates the Notch signaling pathway in MDSCs, potentially enhancing their immunosuppressive capabilities and aiding tumor survival and progression.

To examine how stress hormones influence MDSC-mediated T-cell suppression, we isolated naïve CD8+ T-cells using CD8+ T magnetic beads from naïve C57BL/6 mice. These cells were stimulated with CD3 and CD28 antibodies and co-cultured for 72 hours with MDSCs at ratios ranging from 1:1 to 1:32. Before exposure, MDSCs were pre-treated for 24 hours with stress hormones (10 μM NE, 10 μM EPI, or 10 μM CC) (**Figure 4F**). T-cell suppression was assessed using flow cytometry to measure Granzyme B (GzmB) and Interferon-γ (IFN-γ) expression in CD8^+^ T cells (**Figures 4G, H**; **Supplementary Figures S3A, B**). Flow cytometry analysis demonstrated a significant decrease in the GzmB (**Figure 4I**; **Supplementary Figure S3C**) expression in CD8+ T cells co-cultured with MDSCs treated with NE (*p* = 0.0284), EPI (*p* = 0.0004), and CC (*p* < 0.0001) compared to those co-cultured with vehicle-treated MDSCs across different ratios. This effect was most pronounced in CC-treated MDSCs, with 50% or more T-cell suppression at 1:1, 1:4, 1:8, and 1:32 ratios. Furthermore, results showed a significant decrease in IFN-γ expression (**Figure 4J**; **Supplementary Figure S3D**) in CD8+ T cells co-cultured with MDSCs treated with NE (*p* = 0.0010), EPI (*p* = 0.0036), and CC (*p<* 0.0001) compared to those co-cultured with vehicle-treated MDSCs at different ratios. Similarly, the most pronounced effects were observed in CC-treated MDSCs, with 50% or more T-cell suppression at ratios of 1:2, 1:4, and 1:32. These results suggest stress hormones (NE, EPI, CC), especially CC, enhance the immunosuppressive activity of MDSCs, leading to decreased activation and effector function of CD8^+^ T cells, as shown by lower GzmB and IFN-γ expression. Consequently, chronic stress may facilitate tumor immune evasion by enhancing MDSCs-induced T-cell suppression.

### 3.5 Stress hormones promote the Notch signaling pathway through glycogen synthase kinase 3β in OC cells

**Figure 5:**
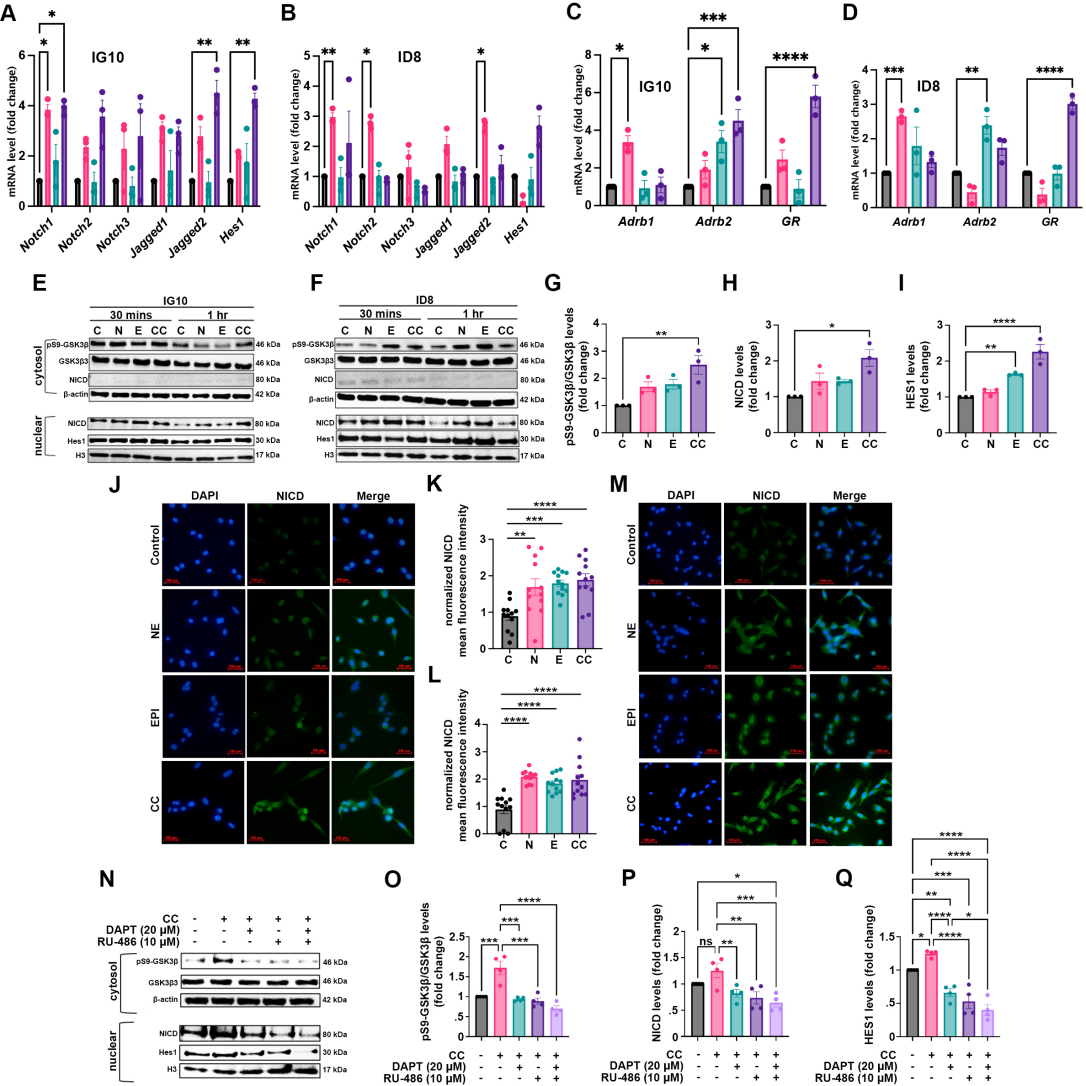
Stress hormones modulate the expression of the Notch signaling pathway proteins in OC cells via GSK3 β. **(A, B)** Notch signaling pathway and **(C, D)** β-adrenergic and glucocorticoid receptors mRNA expression levels following stress hormone treatment in IG10 and ID8 cells for 12 hours. Immunoblots of the Notch signaling pathway protein expression in OC cells **(E)** IG10 and **(F)** ID8 treated with stress hormones at 30 min and 1 hour. Western blot quantification and analysis for pS9-GSK3 β, NICD, and HES1 expression in **(G-I)** IG10 at 1 hour. Representative immunocytochemistry (ICC) images (40X) and quantification of NICD expression in OC cells **(J, K)** IG10 and **(L, M)** ID8 treated with stress hormones at 24 hours; 16 high-power fields were analyzed for each marker with ImageJ software **(N)** Immunoblots of the Notch signaling pathway protein expression in IG10 cells treated with DAPT, RU-486 and CC for 1 hour. Western blot quantification and analysis for **(O)** pS9-GSK3β **(P)** Notch Intracellular Domain (NICD) and **(Q)** HES1 expression in IG10 cells at 1 hour after inhibitory treatments. Statistical analysis was performed using ordinary One-way ANOVA and Two-way ANOVA with Tukey multiple comparison correction (*p < 0.05; **p < 0.01; ***p < 0.001; ****p < 0.0001). Data presented are represented as mean ± SEM of three independent experiments (n=3) for qPCR, western blot and ICC, and four independent experiments (n=4) for inhibitory experiment.

To determine whether stress hormones induce the mRNA levels of Notch signaling members, β-adrenergic, and glucocorticoid receptors in ovarian cancer (OC) cells, we exposed ID8 and IG10 cells to stress hormones (10 μM NE, 10 μM EPI, or 10 μM CC) for 12 hours. qPCR was performed to measure the mRNA levels of several members of the Notch signaling pathway, as well as β-adrenergic and glucocorticoid receptors. CC upregulated mRNA levels of *Notch1* (*p* = 0.0182), *Jagged2* (*p* = 0.0013), and *Hes1* (*p* = 0.0037) in IG10 (**Figure 5A**), while NE increased mRNA levels of *Notch1* (*p* = 0.0058), *Notch2* (*p* = 0.0218), and *Jagged2* (*p* = 0.0352) in ID8 cells (**Figure 5B**). Additionally, stress hormones upregulated the genes for β-adrenergic (*Adrb1* and *Adrb2*) and glucocorticoid (*GR*) receptors through their respective ligands (NE, EPI, and CC) in ID8 and IG10 OC cells (**Figures 5C, D**). These findings indicate a novel role for stress hormones in activating the Notch signaling in ovarian cancer (OC) cells, a pathway essential for regulating cell proliferation, differentiation, and survival.

To dissect the mechanisms by which stress hormones modulate the Notch signaling pathway in OC cells to induce the disease progression, we treated ID8 and IG10 cell lines with stress hormones (10 μM NE, 10 μM EPI, or 10 μM CC) for 5, 15, 30 minutes and 1 hour prior to protein extraction (cytosol and nuclear) and Western blotting (**Figures 5E, F**). Our results showed that CC increased the levels of pS9-GSK3β (*p* = 0.0077) (**Figure 5G**) in the cytosol and NICD (*p* = 0.0107) (**Figure 5H**) and HES1 (*p* = 0.0001) (**Figure 5I**) in the nucleus of IG10 cells at 1 hour, although no changes were found at 5, 15 and 30 minutes (**Supplementary Figures S4A-D**); For ID8, results showed that NE (*p* = 0.0019) and EPI (*p* = 0.0271) increased the levels of pS9-GSK3β at 15 minutes after exposure, but there were no changes in NICD and HES1 levels. Also, non-significant changes were observed at other time points. (**Supplementary Figures S5A-E**). Furthermore, to investigate the effects of stress hormones on the Notch pathway in OC cells, we detected NICD in ID8 and IG10 cells using immunocytochemistry (ICC). We also observed a significant increase in NICD in both IG10 and ID8 cells exposed to stress hormones at 24 and 48 hours (**Figures 5J–M**; **Supplementary Figures S6A-D**). Stress hormone-treated cells consistently exhibited higher levels of NICD expression, with the most pronounced effects observed at 24 hrs in IG10 and ID8 cells. Additionally, we conducted bioinformatics analyses using the STRING platform to explore potential interactions and relationships between the Notch signaling and GSK3β. These analyses revealed interactive networks among proteins of the Notch signaling pathway, including Notch1 and GSK3β (**Supplementary Figure S7A**). We then utilized the GO biological processes database to identify potential pathways related to these interactions (**Supplementary Figure S7B**). The top findings from these analyses included cell communication, regulation of cellular responses to stress, signal transduction, and cancer-related pathways.

To confirm the role of CC in these interactions, we examined the mechanistic effects of inhibiting NICD and glucocorticoid receptors using DAPT ( γ-secretase inhibitor; 20 μM) and RU-486 (glucocorticoid receptor antagonist; 10 μM), respectively, separately and in combination, on IG10 cells. After exposure to these inhibitors, we treated the cells with 10 μM CC for an additional hour and measured levels of pS9-GSK3β, GSK3β, NICD, and the downstream target HES1. Our results showed that treatment with RU-486 significantly decreased the expression of pS9-GSK3β (*p* = 0.0002), NICD (*p* = 0.0020), and HES1 (*p* < 0.0001) in IG10 cells (**Figures 5N–Q**). In addition, treatment with DAPT reduced the expression of these proteins, leading to decreased levels of pS9-GSK3β (*p* = 0.0003), NICD (*p* = 0.0082), and HES1 (*p* < 0.0001). Also, combinatorial therapy using DAPT and RU-486 resulted in a significant reduction in the expression of pS9-GSK3β (*p* < 0.0001), NICD (*p* = 0.0005), and HES1 (*p* < 0.0001) compared to either RU-486 or DAPT alone, effectively blocking CC-induced effects in these cells. Our data suggest that stress hormones facilitate OC progression, potentially by activating Notch-dependent signaling. Furthermore, these findings indicate a novel role for stress hormones, particularly corticosterone, in regulating the Notch signaling pathway in OC cells by inhibiting GSK3β activity. This inhibition is broadly linked to cancer progression and metastasis due to its promotion of the upregulation and expression of the downstream target, HES1.

### 3.6 Chronic restraint stress induces the Notch signaling pathway in tumors from OC-bearing mice

**Figure 6:**
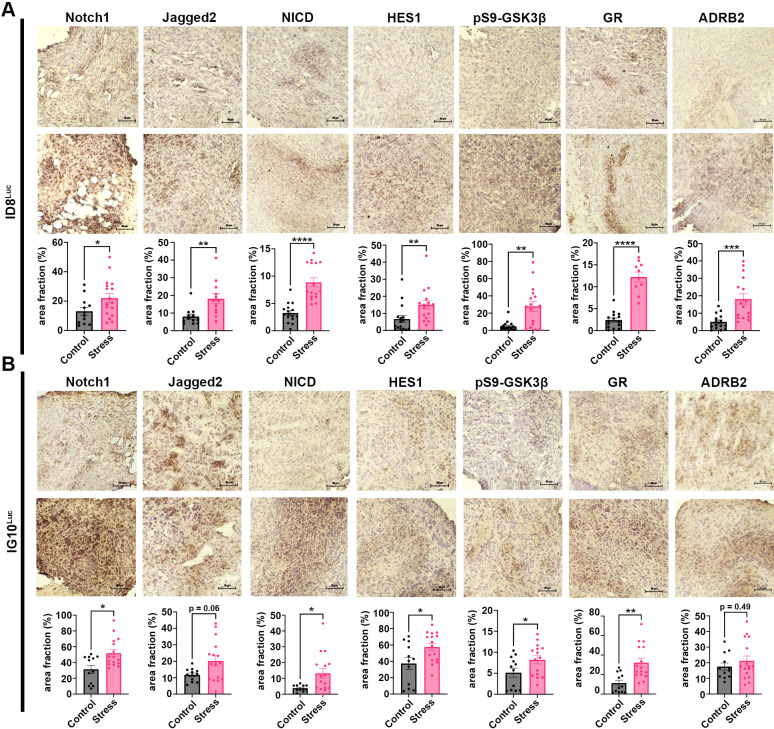
Chronic restraint stress induces the activation of the Notch signaling pathway in tumors from OC-bearing mice subjected to restraint stress. Representative immunohistochemistry (IHC) images (40X) and quantification for Notch signaling pathway members, β-adrenergic, glucocorticoid receptors and pS9-GSK3β expression in tumors from ovarian tumor-bearing mice subjected to restraint stress at week 4, **(A)** ID8^Luc^ (N = 4) and **(B)** IG10^Luc^ (N = 3-4). Statistical analysis was performed using the Mann-Whitney test. Data presented are represented as mean ± SEM. (*p < 0.05; **p < 0.01; ***p < 0.001; ****p < 0.0001).

To investigate the biological impact of chronic restraint stress on the Notch pathway in ovarian tumors, we assessed the expression of Notch pathway members (Notch1, Jagged2, NICD, HES1), β-adrenergic (ADRB2) receptors, glucocorticoid (GR) receptors, and pS9-GSK3β in tumors from both syngeneic mouse models (ID8^Luc^ and IG10^Luc^) using IHC. Our results indicated the induction of Notch1 (*p* = 0.0093), Jagged2 (*p* = 0.0023), NICD (*p* < 0.0001), HES1 (*p* = 0.0004), GR (*p* < 0.0001), ADRB2 (*p* = 0.0002), and pS9-GSK3β (*p* = 0.0066) in tumors from ID8^Luc^-bearing mice subjected to restraint stress (**Figure 6A**). Furthermore, similar results were noted in the IG10^Luc^ model as Notch1 (*p* = 0.0373), NICD (*p* = 0.0198), HES1 (*p* = 0.0331), GR (*p* = 0.0015), and pS9-GSK3β (*p* = 0.0469) were upregulated. Although Jagged2 (*p* = 0.0661) and ADRB2 (*p* = 0.4995) levels increased in response to chronic stress, these findings were not statistically significant in this model. (**Figure 6B**). Together, these results suggest that chronic stress-associated processes activate Notch-dependent signaling pathways, potentially contributing to an immunosuppressive TME and disease progression in OC.

## 4 Discussion

The findings of this study demonstrate that chronic restraint stress accelerates the progression of OC *via* several interrelated mechanisms. These mechanisms include the infiltration of MDSCs, as well as the activation of the Notch signaling pathway in these cells. These results highlight the complex interplay between stress hormones, immunity, and tumor biology, thereby enhancing our understanding of how stress contributes to ovarian cancer progression. As previous studies have indicated, chronic restraint stress triggers the sympathetic nervous system (SNS) and the hypothalamic-pituitary-adrenocortical (HPA) axis (6, 33, 41, 42). This activation leads to an inflammatory environment that progressively induces cancer progression, as suggested by this study and others (32, 33, 41, 43). Further research has associated HPA and SNS dysregulation with various pro-tumoral pathways in cancer cells, including JAK/STAT3, MAPK, PI3K/Akt, WNT/β-catenin, NFκB, and immune system modulation (44–46). Our data also show a significant accumulation of MDSCs in the bone marrow, along with infiltration into the OC TME in mice subjected to chronic restraint stress. This aligns with previous studies that indicate chronic stress promotes the accumulation and immunosuppressive role of MDSCs in various cancers, facilitating immune escape, resistance to treatment, and metastasis (15–18).

The observed enrichment of PMN-MDSCs, along with a decline in M-MDSCs, in mice subjected to chronic restraint stress suggests a shift in MDSCs polarization due to chronic stress. Chronic stress leads to the sustained release of catecholamines and glucocorticoids, thereby inducing the upregulation of inflammatory mediators such as IL-6, G-CSF, IL-1β, and CCL2, as demonstrated by our study and others. (32, 47, 48). These mediators foster an inflammatory environment that promotes the generation, systemic buildup, and migration of MDSCs, and shifts MDSCs into PMN-MDSCs. (49–51). This polarization increases the immunosuppressive ability of MDSCs, as PMN-MDSCs have shown an inhibitory effect on T cell function and enhanced metastasis (17, 52, 53). Such mechanisms of immune evasion may contribute to the accelerated tumor progression seen in mice subjected to chronic restraint stress. Furthermore, the chronic stress-induced influx of MDSCs into tumors corresponds with established roles of glucocorticoids and catecholamines in modulating immune cell trafficking and activity (8, 17, 32, 54, 55).

Additionally, our study shows that daily restraint stress significantly changes the immune cell composition in the ovarian tumor microenvironment, marked by greater infiltration of F4/80^+^ macrophages and reduced presence of CD4^+^ and CD8^+^ T cells. The observed increase in F4/80^+^ macrophages aligns with previous studies indicating that chronic stress can enhance macrophage infiltration into tumors, including ovarian cancer (33, 56–58). These macrophages can release immunosuppressive cytokines that inhibit the regulatory and cytotoxic functions of CD4^+^ and CD8^+^ T cells, thus facilitating tumor growth (59, 60). At the same time, the decrease in CD4^+^ and CD8^+^ T cell populations observed is consistent with reports indicating that chronic stress negatively affects T cell-mediated immunity and induces T cell exhaustion (32, 61–63). All these events can be impacted by increased MDSC infiltration under chronic stress, as evidence indicates that stress hormones in the TME promote immunosuppressive cell populations, such as MDSC or tumor-associated macrophages, which suppress T cell activity and contribute to an immune permissive environment that can enhance tumor progression (58, 64). Thus, it is crucial to emphasize our findings indicating that increased corticosterone levels in mice subjected to chronic stress further reinforces the hypothesis that stress hormones might mediate MDSCs recruitment, survival, immunosuppressive activity, and affect the immune cell composition in the OC TME. Furthermore, the increased expression of Notch signaling pathway members in MDSCs exposed to stress hormones suggests a molecular mechanism by which chronic stress enhances MDSCs-mediated immunosuppression in the OC TME.

Collectively, these findings suggest that chronic restraint stress alters the cellular composition of the ovarian tumor microenvironment and reprograms immune function at a systemic level. This broader immune dysregulation reflects a state of maladaptive immunity, where sustained neuroendocrine activation reprograms immune function from a protective to a tumor-promoting phenotype (9, 54, 65)). Our findings demonstrate that both chronic stress and tumors independently increase the frequency of PMN-MDSCs in the bone marrow, while their combination further amplifies this effect, suggesting a synergistic relationship between stress and tumor-derived factors in driving MDSC expansion (15–17). The increase in PMN-MDSCs, along with the observed decrease in T cell populations and the enrichment of immunosuppressive macrophages, supports the notion that chronic stress creates an immune environment that could promote tumor immune escape. (33, 56, 63). Mechanistically, maladaptive immunity arises when persistent activation of the SNS and HPA axis alters hematopoietic output and immune polarization, shifting immune responses toward chronic inflammation and suppression rather than protection (42, 63). Our group and others have shown that chronic exposure to glucocorticoids and catecholamines promotes the expansion and activation of MDSCs and tumor-associated macrophages while impairing cytotoxic T cell surveillance, resulting in systemic and local immunosuppression (18, 32, 61). Such maladaptive reprogramming represents a pathological deviation from the transient, adaptive stress responses that generally serve to restore homeostasis (65, 66). Consequently, the interplay between chronic stress, MDSCs enrichment, and tumor-driven inflammation establishes a self-perpetuating feedback loop that reinforces immune dysfunction and accelerates ovarian cancer progression (9, 33, 41, 58).

Similarly, our results show that stress hormones, particularly corticosterone, enhance the Notch signaling pathway in OC cells. These findings are important due to the established role of Notch signaling in promoting tumor cell proliferation, survival, and metastasis (10, 22, 23, 67, 68). However, the mechanisms of how chronic stress and stress hormones induce this activation are not well known. Our study highlights the role of Glycogen Synthase Kinase 3β (GSK3β) in regulating Notch signaling in response to stress hormones in OC, inducing tumor cell proliferation. Corticosterone-induced inhibition of GSK3β, through its phosphorylation at serine 9, a known negative regulator of Notch signaling, enhances the activation of this pathway by facilitating the NICD translocation to the nucleus and increasing the upregulation and expression of the main downstream target, HES1, which has been widely linked to cancer progression and proliferation (69–71). These studies demonstrate that GSK3β plays an important role in controlling the stability and activity of NICD by phosphorylating specific serine residues on its C-terminal domain (72, 73). Further supporting this mechanistic pathway, blocking glucocorticoid receptors and NICD effectively stops the changes induced by corticosterone in GSK3β and Notch signaling in OC cells. These findings contribute to the growing evidence linking GSK3β dysregulation to cancer progression through the Notch signaling pathway (26, 74, 75). Additionally, these findings reveal novel mechanisms by which chronic stress or stress hormones can modulate GSK3β activity, promoting OC progression.

The dual role of stress hormones in modulating MDSC biology and activating the Notch signaling pathway in MDSC and ovarian tumors underscores the significant impact of chronic stress on the OC TME. Chronic stress-induced MDSCs enrichment and Notch activation in both MDSCs and OC cells may work synergistically to suppress anti-tumor immunity, promote tumor cell proliferation, and facilitate metastasis. Additionally, the upregulation of β-adrenergic and glucocorticoid receptors may further amplify the effects of stress hormones, creating a feedback loop that enhances tumor progression. Interestingly, the observed differences in the responses of ID8 and IG10 to stress hormones during *in vitro* experiments may arise from a time-dependent effect in activating these pathways, as we noted that NICD expression increased at 24- and 48-hours post-treatment. In addition, as observed in the *in vivo* experiments, both models exhibited similar response patterns since restraint stress leads to sustained activation of the HPA and SNS.

**Figure 7:**
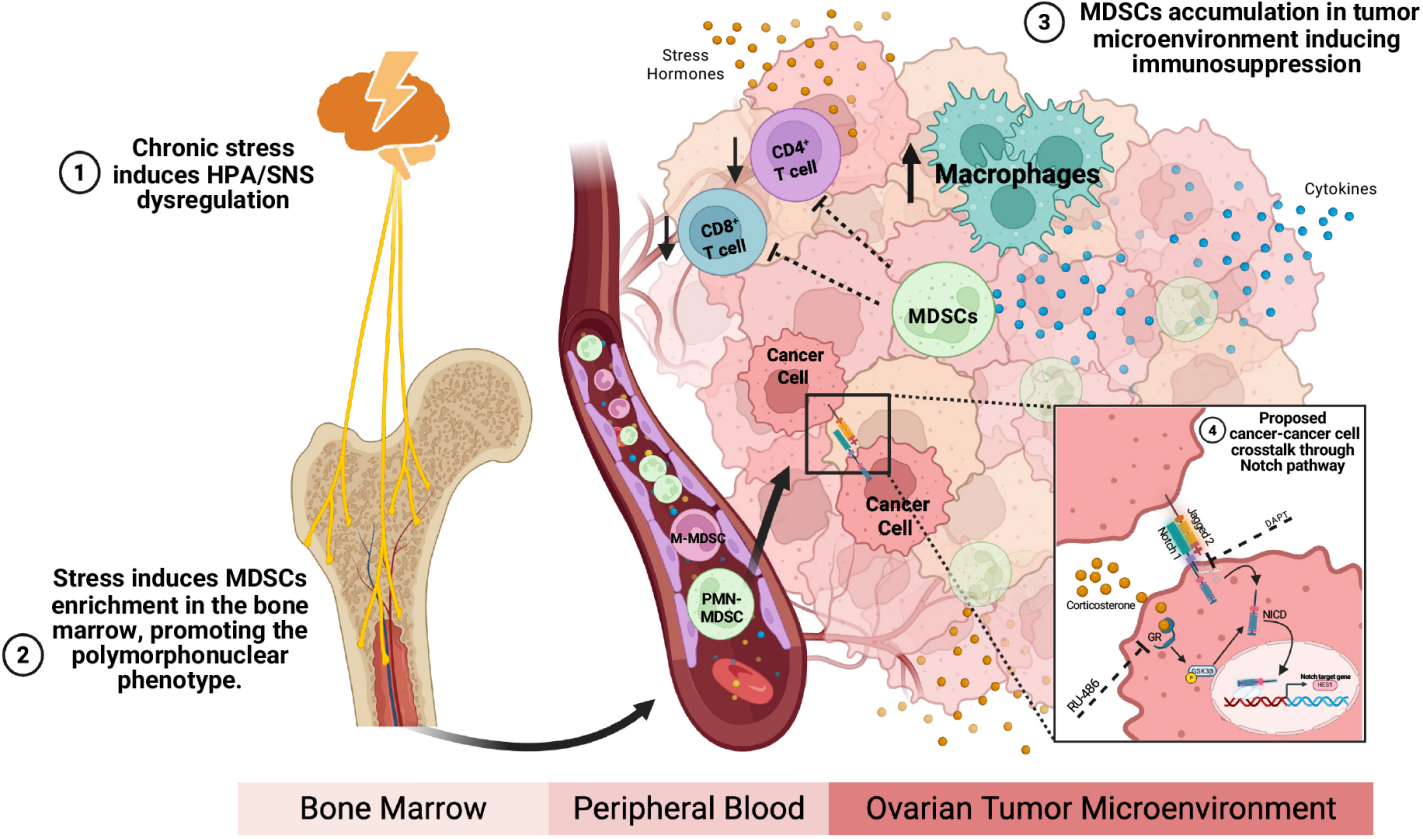
Graphical summary. Proposed model of how chronic stress and the subsequent release of stress hormones contribute to the progression of ovarian cancer progression through MDSCs enrichment in the bone marrow and their subsequent accumulation, polarization into PMN-MDCSs and immunosuppression within the ovarian tumor microenvironment. Furthermore, the potential for ovarian cancer cells to engage in crosstalk through the Notch signaling pathway and GSK3β is also considered.

From a clinical perspective, these findings carry implications for managing chronic stress and its associated effects in cancer patients, particularly those with OC. While this study offers valuable and novel insights into the mechanisms of chronic stress-induced OC progression, future research should also evaluate the effectiveness of targeting MDSCs biology, inhibiting the Notch signaling pathway, and/or blocking glucocorticoid and adrenergic receptors in preclinical models to assess their therapeutic potential in chronic stress-induced OC progression. In summary, this study highlights the role of chronic stress in driving OC progression through MDSCs enrichment, polarization, infiltration, and activation of the Notch signaling pathway in MDSCs and OC. Additionally, our results suggest that corticosterone activates the Notch signaling pathway through GSK3β phosphorylation, resulting in its inactivation and promoting OC progression *via* NICD translocation to the nucleus and HES1 upregulation in OC cells (**Figure 7**). These findings underscore the importance of addressing the effects of chronic stress in patients with OC and support the development of behavioral interventions and therapeutic strategies targeting the stress hormone-immunity-tumor axis.

## Data Availability

The raw data supporting the conclusions of this article will be made available by the authors, without undue reservation.

## Supplementary material

Supplementary Figure 1 MDSCs flow cytometry gating.

Supplementary Figure 2 Experimental design and representative panels for MDSCs in bone marrow.

Supplementary Figure 3 T-cell flow cytometry gating.

Supplementary Figure 4 IG10 western blot quantification.

Supplementary Figure 5 ID8 western blot quantification.

Supplementary Figure 6 Stress hormones induce NICD expression in OC cells at 48 hours.

Supplementary Figure 7 Bioinformatic analysis depicting the potential interaction between the Notch signaling pathway and GSK3 β..

Supplementary Table 1 Cytokines/chemokines data from the Milliplex assay from the serum of OC-bearing mice.

Supplementary Table 2 Primer sequences for qPCR used in this study.

Supplementary Table 3 Antibodies used in this study.
