# Supplementary material for: Chronic stress drives ovarian cancer progression via myeloid-derived suppressor cells infiltration and Notch signaling pathway activation
Source: Front Immunol. 2025 Dec 19;16:1593299. doi: 10.3389/fimmu.2025.1593299 (PMC12757280; doi:10.3389/fimmu.2025.1593299)
Supplement: Supplementary Figure 1 — MDSCs flow cytometry gating. [file DataSheet1.pdf]

Chronic stress drives ovarian cancer progression via Myeloid-Derived Suppressor Cells infiltration and Notch signaling pathway activation

Yadiel A. Rivera-López, Alanis P. Torres-Rosado, Jaydiel A. Casiano-Martínez, Luinet L. Meléndez-Rodríguez, Raian Imad-Hamad, Sofía M. Hernández-Carrasquillo, Luis M. Rivera-Pérez, Melanie Ortiz-León, Orlando I. Torres-Rodríguez, Alexandra N. Aquino-Acevedo, Yesenia Castillo-Ocampo, Grace Duffey, Jaileene Pérez-Morales, Mary K. Townsend, Lauren C. Peres, Paulo C. Rodriguez, Shelley S. Tworoger, and Guillermo N. Armaiz-Pena

Supplementary Material

Supplementary Figure 1

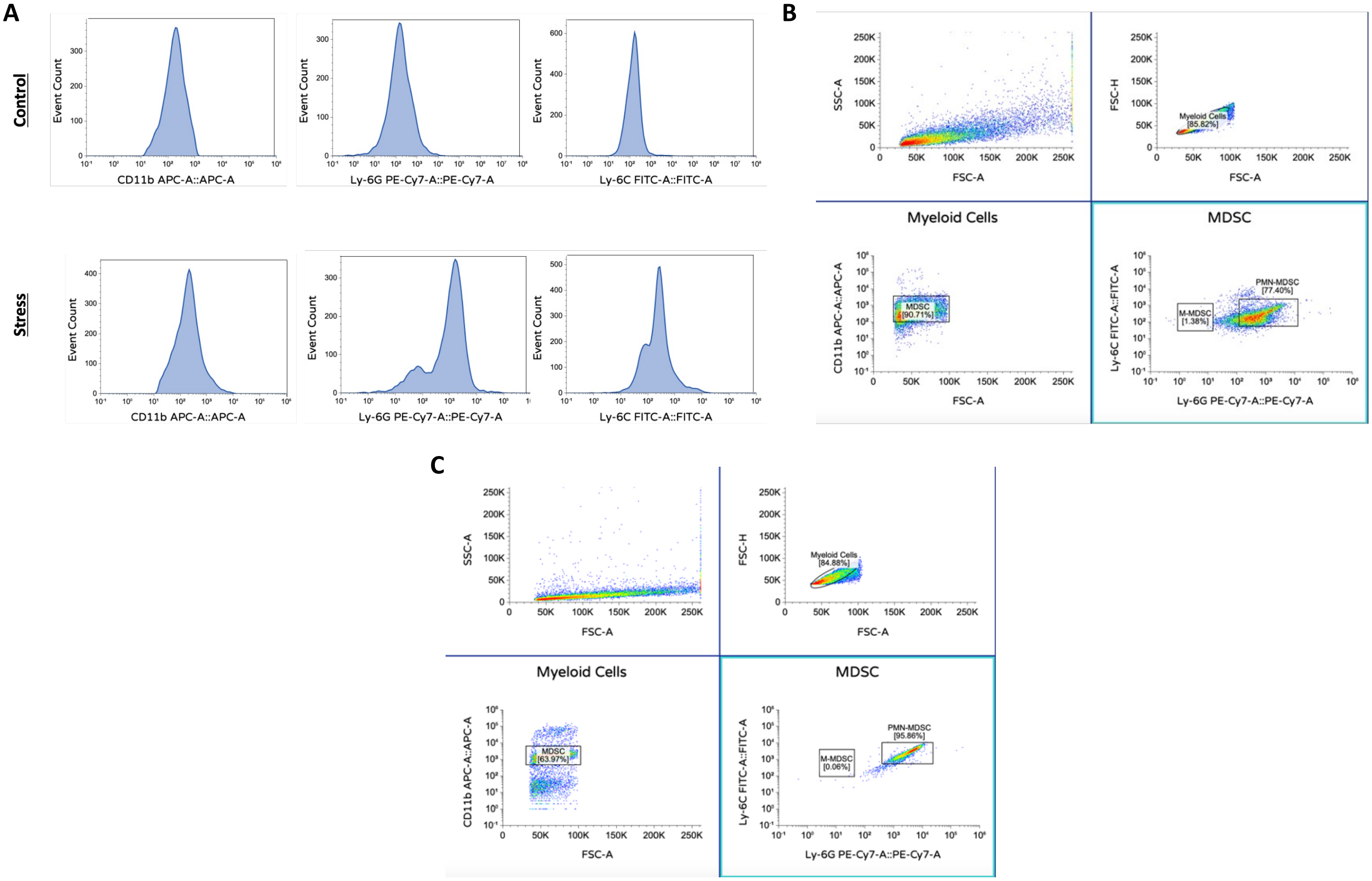

Figure S1. MDSCs flow cytometry gating. (A) Histograms of CD11b$^+$, Ly-6C$^+$ and Ly-6G$^+$ staining distribution in tumors for control and stress groups. Gating strategies for MDSCs in **(B)** ID8^{Luc} and **(C)** IG10^{Luc}.

Supplementary Figure 2

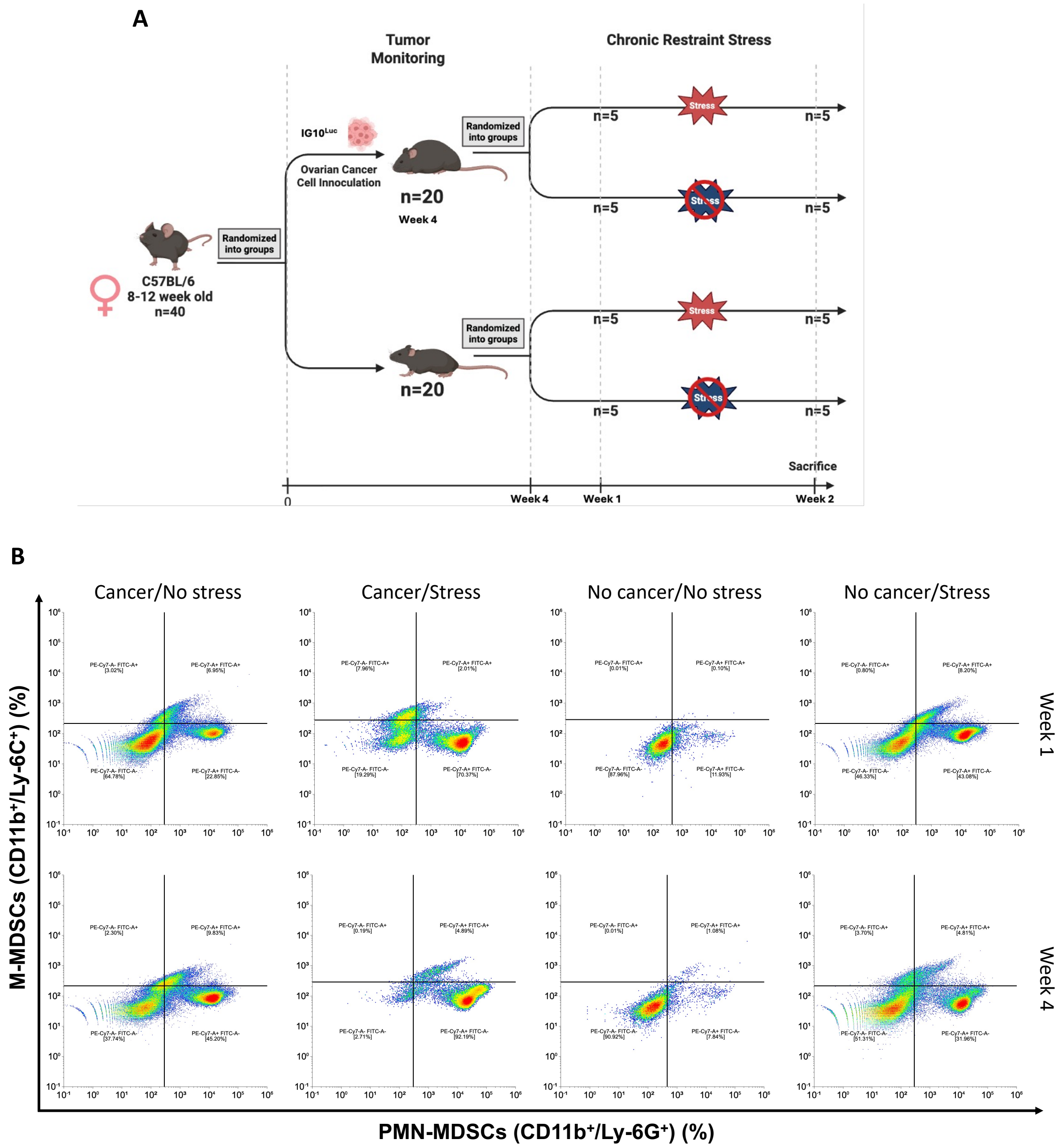

Figure S2. Experimental design and representative panels for MDSCs in bone marrow. (A) Experimental design, which includes the following groups: cancer/no stress, no cancer/stress, no cancer/no stress, and cancer/stress. Created in BioRender.com. **(B)** Representative panels showing cellular expression of polymorphonuclear (PMN)-MDSCs (CD11b+/Ly-6G+) and mononuclear (M)-MDSCs (CD11b+/Ly-6C+) in bone marrow samples, measured by flow cytometry.

Supplementary Figure 3

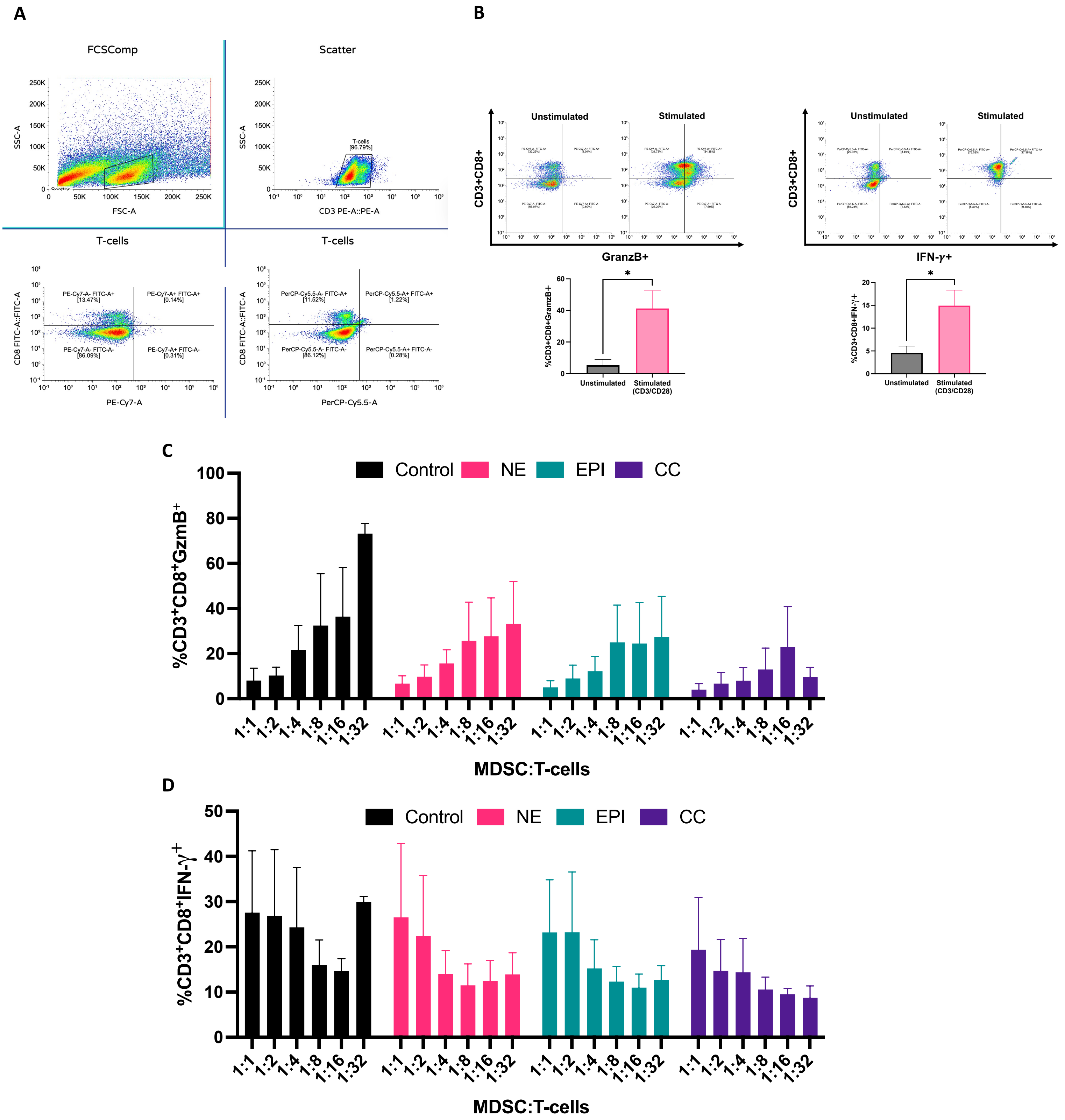

Figure S3. T-cell flow cytometry gating. (A) Gating strategies for CD3$^+$CD8$^+$GzmB$^+$ T-cells and CD3$^+$CD8$^+$IFN-γ$^+$ T-cells. **(B)** MDSCs:T-cells suppression assay controls. Raw cellular expression of **(C)** GzmB+ and **(D)** IFN-γ+ in CD8+ T-cells co-cultured with MDSCs at different ratios, measured by flow cytometry.

Supplementary Figure 4

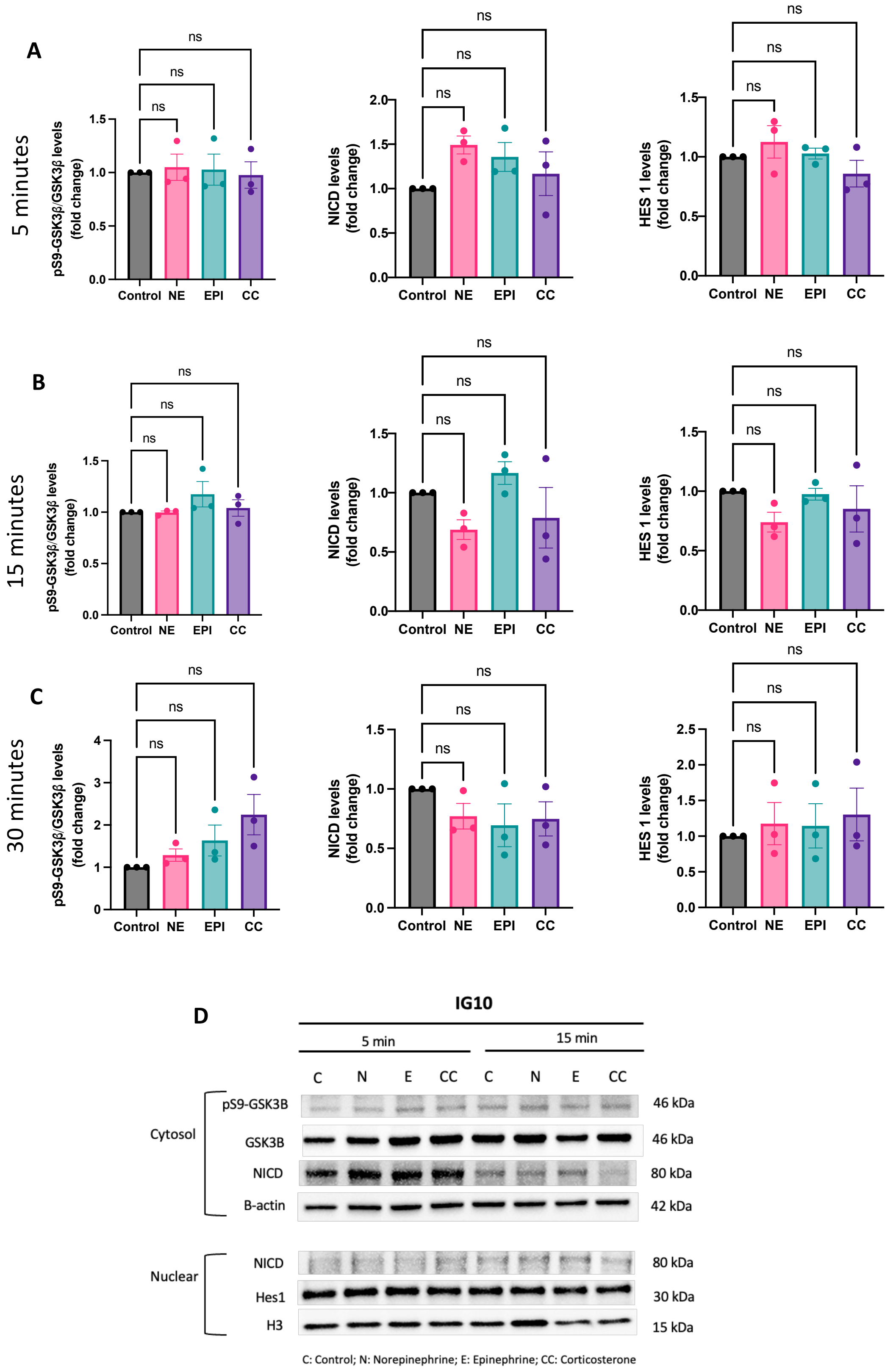

Figure S4. IG10 western blot quantification. Western blots quantification for pS9-GSK3β, NICD and HES1 expression in IG10 cells at **(A)** 5 minutes, **(B)** 15 minutes and **(C)** 30 minutes. **(D)** Immunoblots of the Notch signaling pathway protein expression in IG10 ovarian cancer cells treated with stress hormones at 5 min and 15 min. Statistical analysis was performed using ordinary One-way ANOVA (ns = no significance). Data presented are represented as mean ± SEM of three independent experiments (n=3).

Supplementary Figure 5

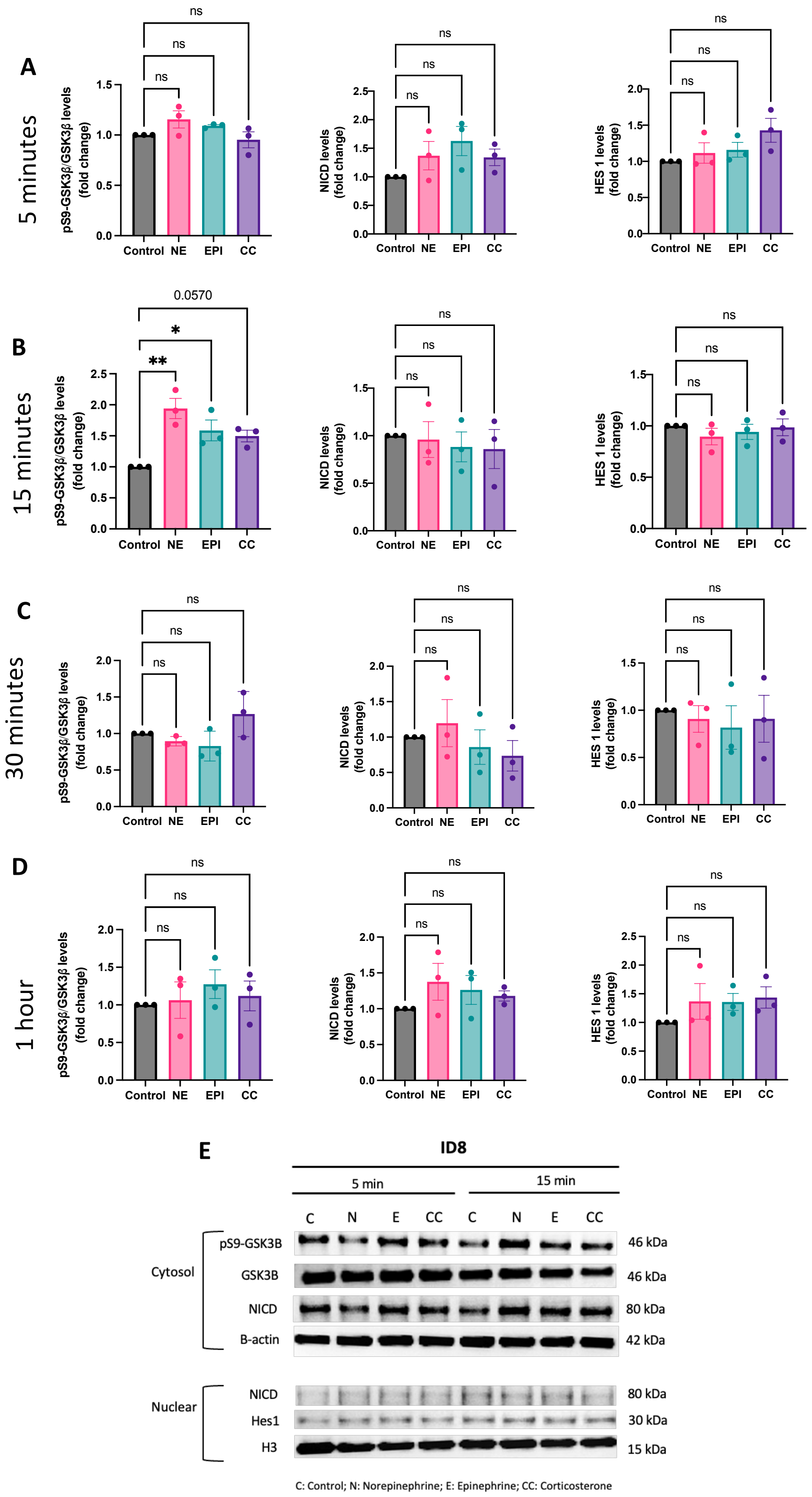

Figure S5. ID8 western blot quantification. Western blots quantification for pS9-GSK3β, NICD and HES1 expression in ID8 cells at **(A)** 5 minutes, **(B)** 15 minutes**, (C)** 30 minutes and **(D)** 1 hour. **(E)** Immunoblots of the Notch signaling pathway protein expression in ID8 ovarian cancer cells treated with stress hormones at 5 min and 15 min. Statistical analysis was performed using ordinary One-way ANOVA (ns = no significance; *p<0.05; **p<0.01). Data presented are represented as mean ± SEM of three independent experiments (n=3).

Supplementary Figure 6

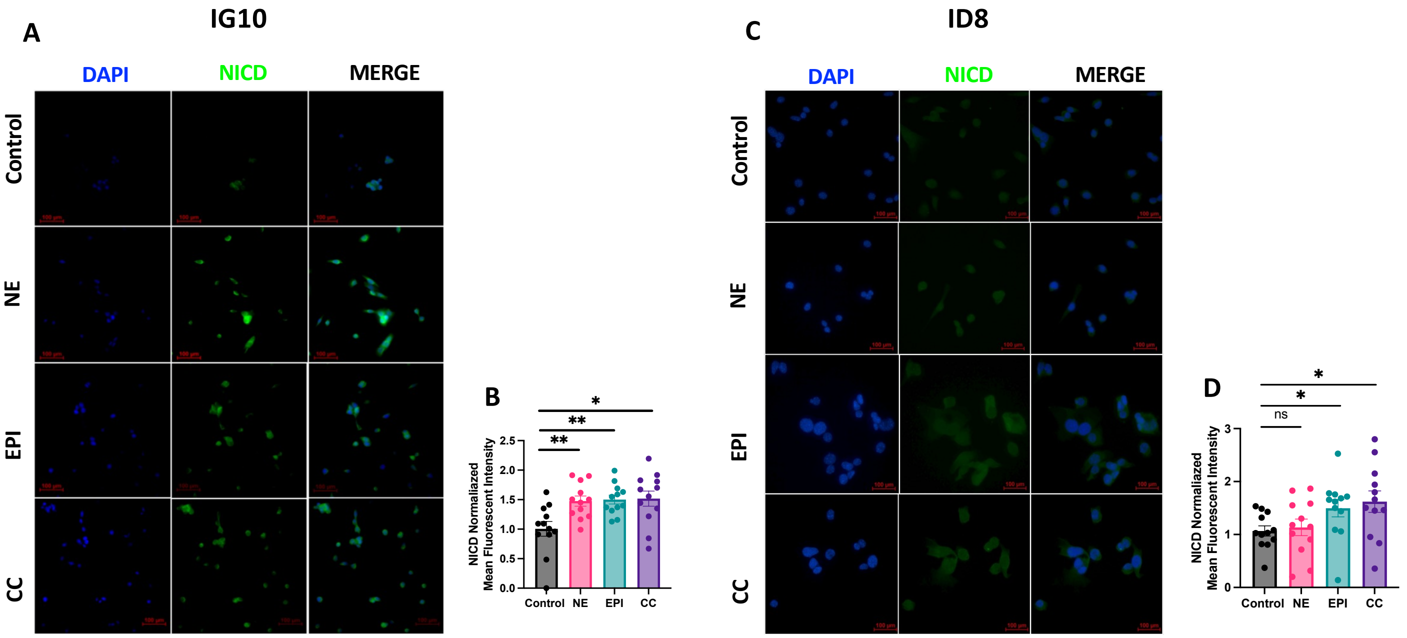

Figure S6. Stress hormones induce NICD expression in OC cells at 48 hours. Representative immunocytochemistry (ICC) images (40X) and quantification of NICD expression in OC cells **(A-B)** IG10 and **(C-D)** ID8 treated with stress hormones at 48 hours. Statistical analysis was performed using ordinary one-way ANOVA (ns = no significance; **p*<0.05; ***p*<0.01). Data presented are represented as mean ± SEM of three independent experiments (n=3).

Supplementary Figure 7

A

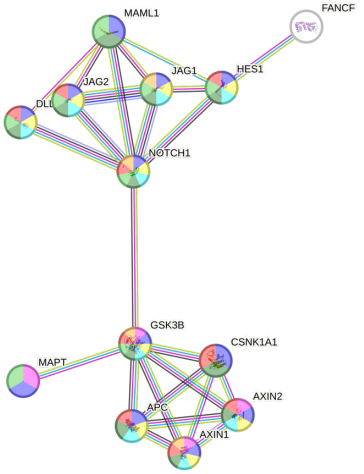

B

#color	category	term ID	term description
yellow	KEGG	hsa05224	Breast cancer
cyan	KEGG	hsa05200	Pathways in cancer
blue	GO Process	GO:0007154	Cell communication
magenta	GO Process	GO:0080135	Regulation of cellular response to stress
darkgreen	Reactome	HSA-162582	Signal Transduction
orange	TISSUES	BTO:0000180	Cervical carcinoma cell
limegreen	WikiPathways	WP61	Notch signaling pathway
red	WikiPathways	WP4262	Breast cancer pathway

Figure S7. Bioinformatic analysis between Notch signaling pathway and GSK3β. (A) Protein-protein interaction network depicting Notch signaling pathway members modulated by GSK3β using STRING platform. **(B)** Bioinformatic analyses utilizing the GO biological processes based on the protein-protein interaction of the Notch signaling pathway members modulated by GSK3β.

Supplementary Table 1

Table 1. Cytokines/chemokines data from Millipex assay using the serum of OC-bearing mice.

Analyte / Sample	IP-10	KC	LIF	IL-9	IL-1 Beta	GM-CSF	LIX	G-CSF	MIP-1 Alfa	MIP-1 Beta	MCP-1
	pg/ml	pg/ml	pg/ml	pg/ml	pg/ml	pg/ml	pg/ml	pg/ml	pg/ml	pg/ml	pg/ml
IG10 C1 W4	109.78	136.3	<1.07	179.36	<0.2	<0.41	426	1342	<0.38	51	<0.42
IG10 C2 W1	256	195.2	<1.07	38.36	0.54	<0.41	9640	380	<0.38	38.62	17.48
IG10 C2 W4	107.06	46.02	<1.07	193.96	1.98	<0.41	214	446	32.4	44.98	35.48
IG10 C3 W4	240	89.56	<1.07	290	3.28	29.74	2674	1770	60.58	<0.37	41.6
IG10 C4 W1	192.96	179	<1.07	186.68	3.28	6.46	4698	494	23.9	91.4	29.38
IG10 C4 W4	198.32	111.9	<1.07	366	0.96	<0.41	2010	982	15.2	56.74	50.84
IG10 C5 W1	149.28	87.98	<1.07	330	8.16	66.88	2494	660	100.66	116.14	218
IG10 C5 W4	149.08	61.46	<1.07	208	2.6	<0.41	884	1368	202	394	63.12
IG10 S1 W1	198.32	744	80.46	250	63.62	476	10686	1140	32.4	51	90.46
IG10 S2 W1	181.6	306	<1.07	438	80.54	264	8886	838	<0.38	56.74	111.76
IG10 S2 W4	143.2	72.1	<1.07	164.52	46.4	193.96	1268	594	40.68	67.52	47.76
IG10 S3 W1	154.26	132.02	<1.07	149.48	82.3	198.32	5204	876	15.2	53.9	394
IG10 S3 W4	170.38	101.56	<1.07	236	84	43.08	3490	1132	40.68	56.74	175.9
IG10 S4 W1	202	524	<1.07	202	61.44	1.66	10882	764	40.68	82.28	135.48
IG10 S4 W4	250	105.72	<1.07	193.96	130.46	224.8	744	1352	<0.38	62.24	101.66
IG10 S5 W1	204	204	<1.07	118.72	47.08	148.64	11368	376	6.34	>9945.22	114.04
ID8 C1 W1	99.55	69.9	<1.07	74.74	0.48	<0.41	2002	168	<0.38	12.22	<0.42
ID8 C1 W4	134	85.65	<1.07	74.74	<0.2	0.62	5203	377	<0.38	8.12	3.2
ID8 C2 W4	62.4	90.7	<1.07	125	0.27	0.78	187	957	11.95	10.24	<0.42
ID8 C3 W1	118	79.8	1.55	51.5	1.3	0.58	926	391	14.09	38.76	0.83
ID8 C3 W4	84.6	85.53	<1.07	59.36	0.52	<0.41	1000	492	<0.38	25.5	<0.42
ID8 C4 W1	77.35	53.76	<1.07	132	0.72	<0.41	1104	389	7.6	43.45	<0.42
ID8 C4 W4	87.43	544	18.18	59.36	0.63	0.61	468	212	<0.38	12.22	<0.42
ID8 C5 W1	101	115	7.52	43.54	<0.2	<0.41	1541	210	<0.38	<0.37	0.83
ID8 C5 W4	161	131	<1.07	51.5	2	<0.41	1737	917	<0.38	25.5	23.88
ID8 S1 W1	50.56	51.3	<1.07	249	<0.2	51.5	691	202	16.2	<0.37	23.82
ID8 S1 W4	92.96	59.93	<1.07	104	15.36	90.7	1501	264	<0.38	<0.37	25.11
ID8 S2 W1	79.84	38.84	<1.07	51.5	0.48	66.01	1359	335	11.95	8.12	24.69
ID8 S2 W4	96.78	37.25	2.1	96.98	<0.2	85.19	3044	721	<0.38	33.76	24.75
ID8 S3 W1	87.78	44.25	<1.07	82.26	0.48	54.14	3008	243	<0.38	28.37	22.23
ID8 S3 W4	70.23	97.6	<1.07	74.74	12.26	74.64	291	733	<0.38	5.82	3.2
ID8 S4 W1	78.05	54.15	<1.07	27.35	23.53	<0.41	335	247	<0.38	<0.37	<0.42
ID8 S4 W4	78.49	119	<1.07	129	17.09	93.64	237	822	<0.38	19.31	<0.42
ID8 S5 W4	112	30.2	2.04	89.68	25.12	82.41	573	583	<0.38	12.22	0.83

Analyte / Sample	M-CSF	MIG	IL-7	IL-12(p70)	IL-15	IL-10	IL-1 Alfa	IL-12(p40)	IL-17	IL-4	IL-5
	pg/ml	pg/ml	pg/ml	pg/ml	pg/ml	pg/ml	pg/ml	pg/ml	pg/ml	pg/ml	pg/ml
IG10 C1 W4	<0.4	228	<0.47	<0.36	1.28	6.06	26.06	3.52	11.42	<0.62	<0.99
IG10 C2 W1	<0.4	1348	<0.47	<0.36	8.34	1.72	12.58	5.36	6.98	<0.62	12.2
IG10 C2 W4	3.02	324	8.72	4.7	6.42	7.38	54.64	9.56	12.74	<0.62	7.3
IG10 C3 W4	3.02	654	<0.47	6.44	27.16	16.42	105.18	2.56	8.54	<0.62	2.88
IG10 C4 W1	5.28	506	<0.47	10.26	20.7	6.94	167.86	3.7	7.44	<0.62	7.04
IG10 C4 W4	<0.4	107.54	<0.47	3.14	68.88	20.86	6.38	<0.9	22.64	<0.62	<0.99
IG10 C5 W1	15.04	350	2.9	8.3	22.84	12.2	278	8.54	16.6	1.98	8.34
IG10 C5 W4	<0.4	173.62	<0.47	12.34	16.46	14.82	80.02	<0.9	16.6	<0.62	<0.99
IG10 S1 W1	<0.4	476	<0.47	6.44	27.16	15.34	364	9.22	12.74	2.6	19.8
IG10 S2 W1	1.18	1044	<0.47	<0.36	31.52	<0.86	202	3.36	2.68	<0.62	5.78
IG10 S2 W4	<0.4	226	<0.47	19.06	1.28	23.16	76.4	<0.9	12.74	3.84	25.8
IG10 S3 W1	<0.4	570	<0.47	6.44	16.46	45.64	302	4.42	8.76	<0.62	11.5
IG10 S3 W4	4.68	68.86	<0.47	10.26	11.3	106.54	167.86	2.12	13.58	<0.62	<0.99
IG10 S4 W1	6.52	580	<0.47	6.44	30.44	25.64	174.6	2.86	5.5	<0.62	22.18
IG10 S4 W4	<0.4	362	<0.47	<0.36	16.46	58.34	54.64	2.56	2.8	<0.62	3.28
IG10 S5 W1	<0.4	1778	<0.47	1.8	14.38	27.38	208	5.76	11.84	<0.62	6.54
ID8 C1 W1	<0.4	238	<0.47	18.33	<0.29	11	131	1.35	15.05	<0.62	4.85
ID8 C1 W4	<0.4	428	<0.47	<0.36	4.66	2.03	251	2.44	5.39	<0.62	<0.99
ID8 C2 W4	<0.4	49.88	<0.47	3.22	4.17	10.15	45.42	<0.9	5.8	<0.62	1.64
ID8 C3 W1	<0.4	280	<0.47	0.9	5.15	1.66	63.22	1.85	6.87	<0.62	5.4
ID8 C3 W4	<0.4	581	1.36	<0.36	<0.29	1.49	34.57	2.12	10.34	<0.62	3.52
ID8 C4 W1	<0.4	723	<0.47	0.9	28.96	3.25	70.2	3.29	6.5	<0.62	5.4
ID8 C4 W4	<0.4	175	<0.47	<0.36	<0.29	0.86	3.19	<0.9	4.74	<0.62	6.1
ID8 C5 W1	<0.4	454	<0.47	<0.36	<0.29	1.84	38.2	<0.9	4.11	<0.62	3.02
ID8 C5 W4	<0.4	194	<0.47	<0.36	39.33	6.23	34.57	1.2	1.97	<0.62	3.27
ID8 S1 W1	<0.4	263	<0.47	<0.36	2.29	<0.86	16.55	<0.9	1.1	<0.62	3.14
ID8 S1 W4	<0.4	263	<0.47	<0.36	37.16	<0.86	27.32	<0.9	8.26	<0.62	3.46
ID8 S2 W1	17.39	180	<0.47	3.22	6.16	1.66	34.57	1.51	4.82	<0.62	<0.99
ID8 S2 W4	<0.4	240	<0.47	<0.36	<0.29	2.72	135	<0.9	6.87	<0.62	4.31
ID8 S3 W1	0.59	367	<0.47	<0.36	10.35	1.66	139	1.28	3.41	<0.62	3.65
ID8 S3 W4	<0.4	38.82	<0.47	<0.36	<0.29	6.88	6.29	<0.9	<0.96	<0.62	<0.99
ID8 S4 W1	<0.4	460	<0.47	<0.36	12.5	0.86	27.32	2.39	3.34	<0.62	1.64
ID8 S4 W4	<0.4	121	<0.47	<0.36	27.86	3.25	30.95	0.99	4.82	<0.62	1.24
ID8 S5 W4	<0.4	223	51.91	<0.36	354	11.86	20.11	1.35	3.04	<0.62	<0.99

Analyte / Sample	IL-6	IL-2	IFN-Gamma	IL-3	MIP-2	RANTES	VEGF	TNF-Alfa	Eotaxin
	pg/ml	pg/ml	pg/ml	pg/ml	pg/ml	pg/ml	pg/ml	pg/ml	pg/ml
IG10 C1 W4	29.18	2.94	3.36	<1.12	<0.6	26.56	<1.14	<1.42	167.28
IG10 C2 W1	4.82	2.02	2.12	<1.12	50.84	39.54	<1.14	3.34	452
IG10 C2 W4	15.56	2.38	3.56	<1.12	12.04	32.9	<1.14	5	61.48
IG10 C3 W4	13.28	3.12	3.36	<1.12	38.36	29.7	<1.14	13.8	234
IG10 C4 W1	4.08	3.02	1.96	<1.12	38.36	20.54	<1.14	5.58	336
IG10 C4 W4	5.1	3.12	3.56	<1.12	3.18	36.88	<1.14	35.1	216
IG10 C5 W1	7.52	3.52	2.96	<1.12	91.88	22.4	<1.14	8.8	266
IG10 C5 W4	32.92	4.26	2.12	<1.12	1.96	25.28	<1.14	13.38	180.1
IG10 S1 W1	44.02	3.32	0.92	<1.12	32.46	51.58	<1.14	26.36	396
IG10 S2 W1	143.16	2.46	1.46	<1.12	21.5	66.66	<1.14	3	1158
IG10 S2 W4	72.92	1.54	<0.32	<1.12	<0.6	54.98	<1.14	31.3	230
IG10 S3 W1	146.68	1.78	<0.29	<1.12	26.82	54.06	<1.14	24.06	286
IG10 S3 W4	179.46	2.38	<0.29	<1.12	32.46	50.96	<1.14	24.16	350
IG10 S4 W1	146.4	2.28	1.46	<1.12	44.5	39.5	<1.14	4.44	592
IG10 S4 W4	136.56	<0.7	<0.32	<1.12	4.62	56.56	<1.14	27.58	266
IG10 S5 W1	105.08	2.46	1.56	<1.12	26.82	37.54	<1.14	3	606
ID8 C1 W1	1.92	0.93	1.68	<1.12	2.31	6.78	<1.14	2.5	109
ID8 C1 W4	1.92	0.93	1.34	<1.12	8.28	7.45	<1.14	<1.42	207
ID8 C2 W4	0.53	2.62	1.29	<1.12	0.98	5.48	<1.14	3.18	76.99
ID8 C3 W1	1.8	<0.7	1.59	<1.12	25.42	10	<1.14	1.85	118
ID8 C3 W4	6.49	<0.7	<0.32	<1.12	<0.6	3.53	<1.14	<1.42	86.33
ID8 C4 W1	0.53	1.01	0.78	<1.12	8.28	10.69	1.19	1.5	82.99
ID8 C4 W4	350	<0.7	0.94	<1.12	<0.6	7.07	<1.14	<1.42	102
ID8 C5 W1	2.8	0.93	0.83	<1.12	2.31	11.37	<1.14	<1.42	108
ID8 C5 W4	1.25	<0.7	<0.32	<1.12	0.98	19.09	<1.14	<1.42	199
ID8 S1 W1	30.79	1.66	<0.47	<1.12	<0.6	24.52	<1.14	3.99	118
ID8 S1 W4	11.68	<0.7	<0.47	<1.12	0.98	16.89	<1.14	2.49	86.54
ID8 S2 W1	16.28	1.01	<0.36	<1.12	13.41	22.64	<1.14	2.85	133
ID8 S2 W4	24.49	1.1	<0.36	<1.12	0.98	14.1	<1.14	<1.42	143
ID8 S3 W1	11.15	<0.36	0.54	<1.12	8.28	22.75	<1.14	1.67	164
ID8 S3 W4	56.67	<0.7	0.55	<1.12	<0.6	25.45	<1.14	1.54	130
ID8 S4 W1	10.86	<0.7	<0.32	<1.12	<0.6	25.53	<1.14	2.13	106
ID8 S4 W4	21.55	1.1	0.89	<1.12	<0.6	14.44	<1.14	1.88	126
ID8 S5 W4	19.36	1.01	0.44	<1.12	<0.6	24.85	<1.14	1.85	186

Abbreviations: C: Control; S: Stress; W: Week

Supplementary Table 2

Table 2. Primers sequences for qPCR used in this study.

Gene	Forward Primer	Reverse Primer
Notch1	GGT GAA CAA TGT GGA TGC TG	GCA ACA CTT TGG CAG TCT CA
Notch2	GAG GAT GAG GCT TTG CTG TC	GTT CTG CCT GAG GAG GAG TG
Notch3	CTC TGT GGT GAT GCT GGA GA	AAT CAA GTC GCT CCA CTG CT
Jagged1	GGA AGT GGA GGA GGA TGA CA	GTC CAG TTC GGG TGT TTT GT
Jagged2	TCC GAG TAC GCT GTG ATG AG	GGC TTC TTT GCA TTC TTT GC
Hes1	CCG AGC GTG TTG GGG AAA TAC	GTT GAT CTG GGT CAT GCA GTT GG
GR	TGG AAA CCT GCT ATG CTT TGC TC	AAC CGC TGC CAA TTC TGA CTG
Gapdh	ACC ACA GTC CAT GCC ATC AC	TCC ACC ACC CTG TTG CTG TA

Supplementary Table 3

Table 3. Antibodies used in this study.

Antibody	Technique	Company	Catalog Number
**Cleaved Notch1 (NICD)**	Immunohistochemistry<br>Immunocytochemistry	Cell Signaling	4147S
**Anti-Notch1, Notch Intracellular Domain (NICD)**	Western blot	Sigma	07-1232
**pS9-GSK3β**	Western blot	Cell Signaling	9336S
**GSK3β**	Western blot	abcam	ab93926
**HES1**	Western blot<br>Immunohistochemistry	Cell Signaling	11988S
**β-actin**	Western blot	Cell Signaling	3700S
**Histone H3**	Western blot	Cell Signaling	4499S
**Notch1**	Immunohistochemistry	Invitrogen	MA5-32080
**Jagged2**	Immunohistochemistry	Thermo Fisher	PA5-102863
**ADRB2**	Immunohistochemistry	abcam	ab182136
**GR**	Immunohistochemistry	Cell Signaling	12041S
**pS9-GSK3β**	Immunohistochemistry	abcam	ab107166
**CD4**	Immunohistochemistry	Novus Biologicals	NBP2-25191
**CD8α**	Immunohistochemistry	R&D Systems	MAB116
**Anti-F4/80**	Immunohistochemistry	abcam	ab6640
**CD11b**	Immunofluorescence	abcam	ab184308
**Gr-1 (Ly-6G/Ly-6C)**	Immunofluorescence	R&D Systems	MAB1037
**CD11b-APC**	Flow Cytometry	abcam	ab25482
**Ly-6G-PE/Cy7**	Flow Cytometry	abcam	ab25514
**Ly-6C-FITC**	Flow Cytometry	abcam	ab25025
**CD3**	Flow Cytometry	Invitrogen	12-0032-82
**CD8**	Flow Cytometry	Invitrogen	MCD0801
**Granzyme B**	Flow Cytometry	BioLegend	396410
**Interferon-γ**	Flow Cytometry	BioLegend	505822
**Goat Anti-Rabbit IgG (H+L)-HRP Conjugate**	Western blot	Bio-Rad	170-6515
**Goat Anti-Mouse IgG (H+L)-HRP Conjugate**	Western blot	Bio-Rad	170-6516
**Alexa Fluor 488 Goat anti-Rat**	Immunofluorescence	abcam	ab150157
**Alexa Fluor 594 Goat anti-Rabbit**	Immunofluorescence	abcam	ab150080
**Alexa Fluor 488-Conjugated AffiniPure F'(ab') Fragment Goat Anti-Rabbit IgG**	Immunocytochemistry	Jackson ImmunoResearch	111-546-047
**Rat IgG2b kappa Isotype Control (FITC)**	Flow Cytometry	Invitrogen	11-4031-82
**American Hamster IgG Isotype Control (APC)**	Flow Cytometry	Invitrogen	17-4888-82
**PE/Cy7 Rat IgG2a, monoclonal [2A3] - Isotype Control**	Flow Cytometry	abcam	ab253051
**Rat IgG2b kappa Isotype Control (PE)**	Flow Cytometry	Invitrogen	12-4031-82
**PerCP/Cy5.5 Rat IgG2a, kappa Isotype Control**	Flow Cytometry	BioLegend	400532
