# Supplementary material for: Chronic stress drives ovarian cancer progression via myeloid-derived suppressor cells infiltration and Notch signaling pathway activation
Source: Front Immunol. 2025 Dec 19;16:1593299. doi: 10.3389/fimmu.2025.1593299 (PMC12757280; doi:10.3389/fimmu.2025.1593299)
Supplement: Supplementary Table 1 — Cytokines/chemokines data from the Milliplex assay from the serum of OC-bearing mice. [file DataSheet2.pdf]

Whole immunoblots

Immunoblots: IG10-Cytosol

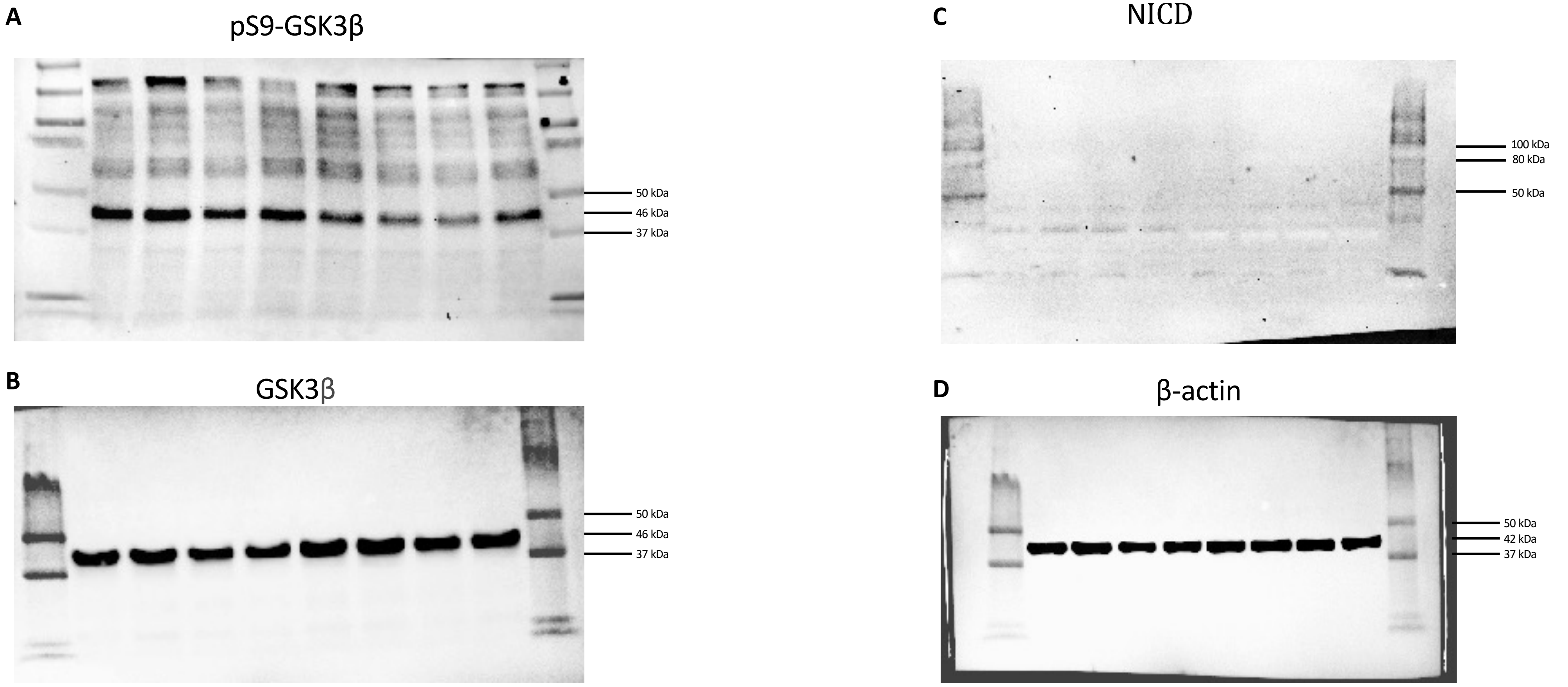

Figure S1. Original Western blot films from cytosol fractionation (**Fig. 5E**) showing pS9-GSK3β **(A)** GSK3β **(B)** NICD **(C)** and B-actin **(D)** expression in IG10 ovarian cancer cells treated with stress hormones (norepinephrine (NE), epinephrine (EPI) or corticosterone (CC)) for 30 minutes and 1 hour.

Immunoblots: IG10-Nuclear

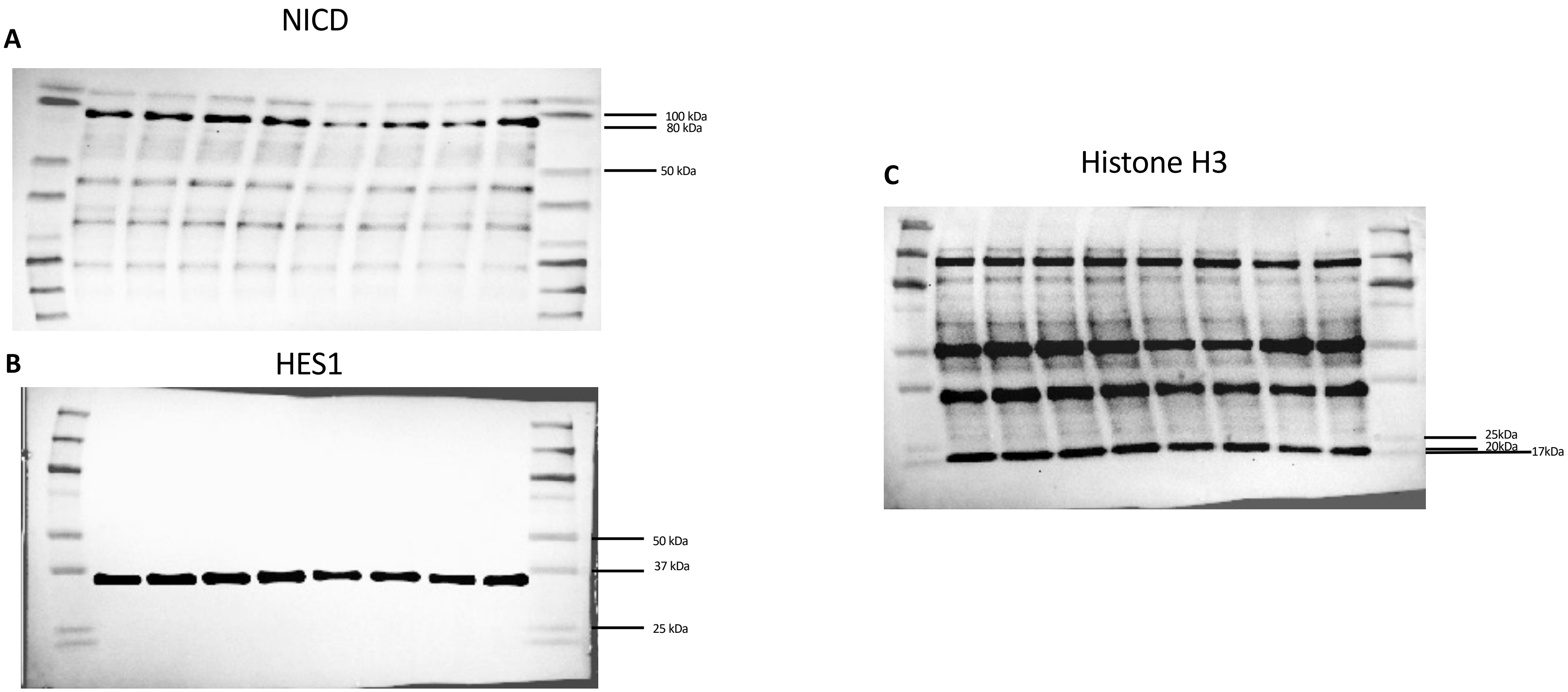

Figure S2. Original Western blot films from nuclear fractionation (**Fig. 5E**) showing NICD **(A)** HES1 **(B)** and Histone (H3) **(C)** expression in IG10 ovarian cancer cells treated with stress hormones (norepinephrine (NE), epinephrine (EPI) or corticosterone (CC)) for 30 minutes and 1 hour.

Immunoblots: ID8-Cytosol

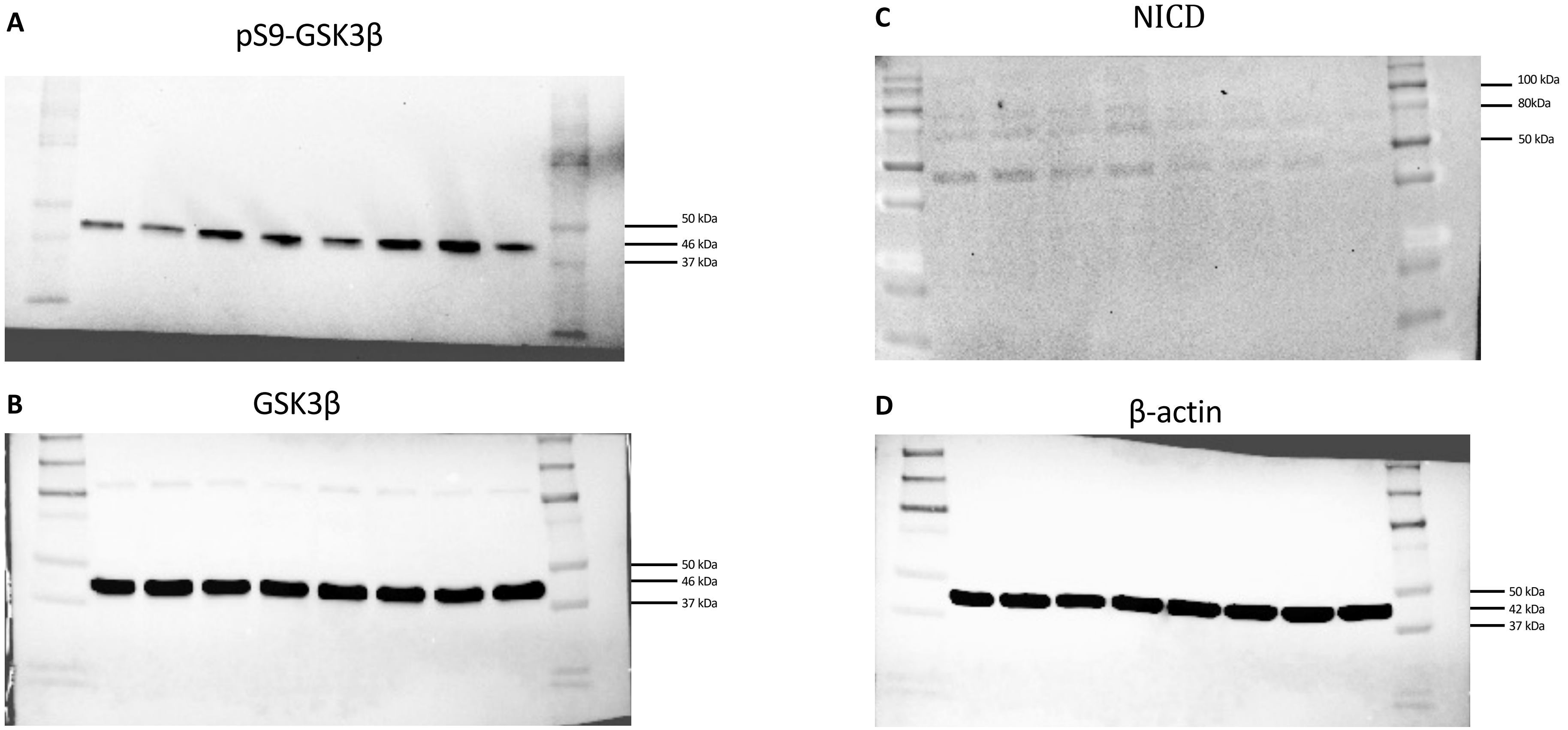

Figure S3. Original Western blot films from cytosol fractionation (**Fig. 5F**) showing pS9-GSK3β **(A)** GSK3β **(B)** NICD **(C)** and B-actin **(D)** expression in ID8 ovarian cancer cells treated with stress hormones (norepinephrine (NE), epinephrine (EPI) or corticosterone (CC)) for 30 minutes and 1 hour.

Immunoblots: ID8-Nuclear

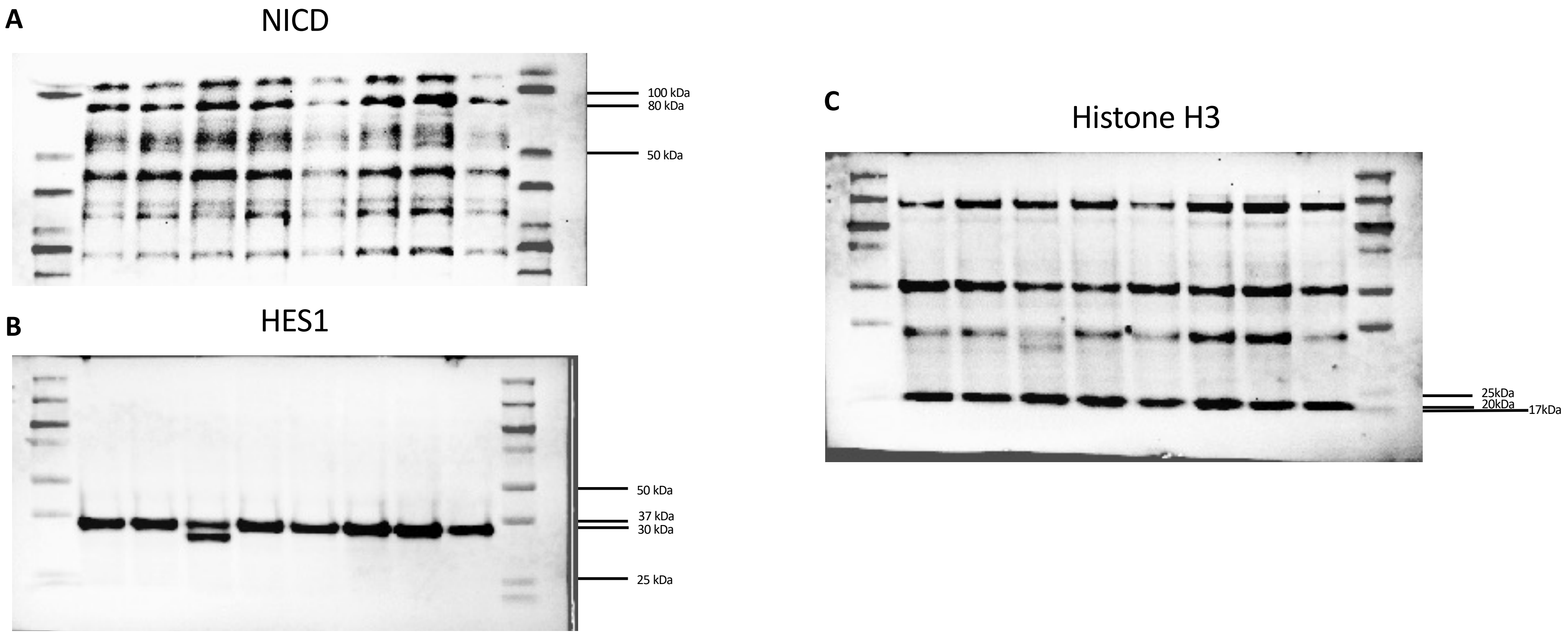

Figure S4. Original Western blot films from nuclear fractionation (**Fig. 5F**) showing NICD **(A)** HES1 **(B)** and Histone (H3) **(C)** expression in ID8 ovarian cancer cells treated with stress hormones (norepinephrine (NE), epinephrine (EPI) or corticosterone (CC)) for 30 minutes and 1 hour.

Immunoblots-Inhibition Experiment: IG10-Cytosol

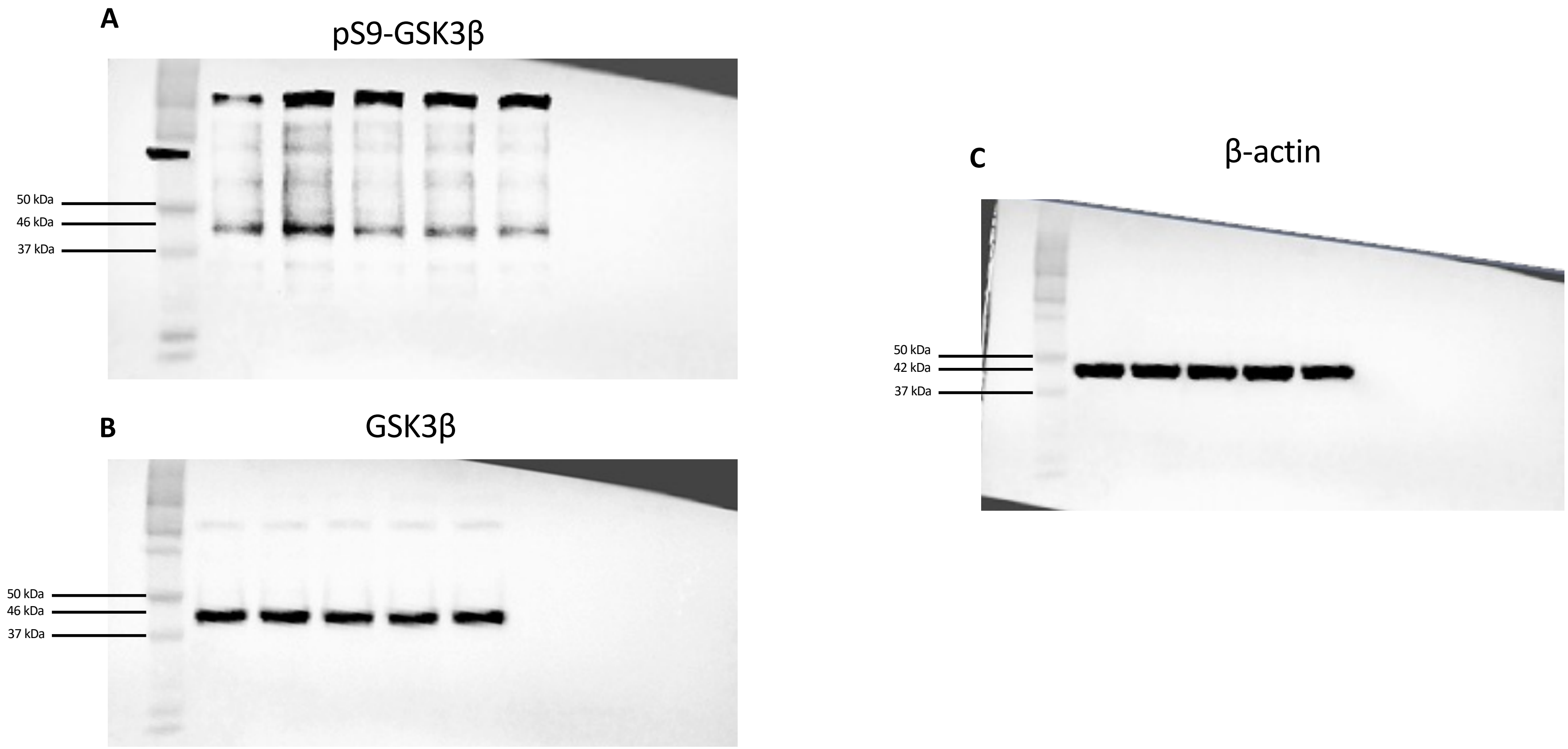

Figure S5. Original Western blot films from cytosol fractionation (**Fig. 5N**) showing pS9-GSK3β **(A)** GSK3β **(B)** and B-actin **(C)** expression in IG10 ovarian cancer cells treated with inhibitors (DAPT and RU-486) for 1 hour and then treated with corticosterone (CC) for 1 hour.

Immunoblots-Inhibition Experiment: IG10-Nuclear

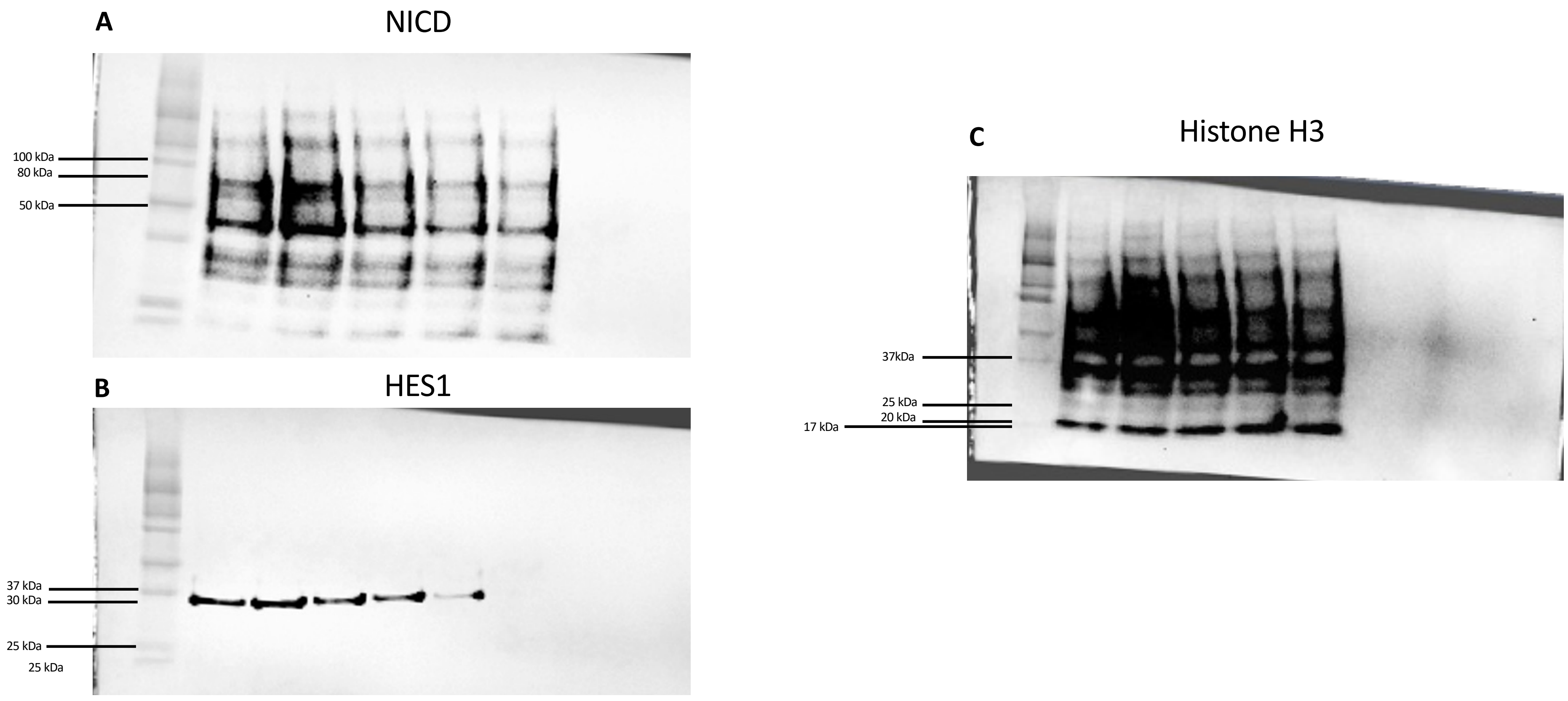

Figure S6. Original Western blot films from nuclear fractionation (**Fig. 5N**) showing NICD **(A)** HES1 **(B)** and Histone (H3) **(C)** expression in IG10 ovarian cancer cells treated with inhibitors (DAPT and RU-486) for 1 hour and then treated with corticosterone (CC) for 1 hour.
